# Structure of the Trehalose-6-phosphate Phosphatase from *Brugia malayi* Reveals Key Design Principles for Anthelmintic Drugs

**DOI:** 10.1371/journal.ppat.1004245

**Published:** 2014-07-03

**Authors:** Jeremiah D. Farelli, Brendan D. Galvin, Zhiru Li, Chunliang Liu, Miyuki Aono, Megan Garland, Olivia E. Hallett, Thomas B. Causey, Alana Ali-Reynolds, Daniel J. Saltzberg, Clotilde K. S. Carlow, Debra Dunaway-Mariano, Karen N. Allen

**Affiliations:** 1 Department of Chemistry, Boston University, Boston, Massachusetts, United States of America; 2 New England Biolabs, Division of Parasitology, Ipswich, Massachusetts, United States of America; 3 Department of Chemistry and Chemical Biology, University of New Mexico, Albuquerque, New Mexico, United States of America; Rush University Medical Center, United States of America

## Abstract

Parasitic nematodes are responsible for devastating illnesses that plague many of the world's poorest populations indigenous to the tropical areas of developing nations. Among these diseases is lymphatic filariasis, a major cause of permanent and long-term disability. Proteins essential to nematodes that do not have mammalian counterparts represent targets for therapeutic inhibitor discovery. One promising target is trehalose-6-phosphate phosphatase (T6PP) from *Brugia malayi*. In the model nematode *Caenorhabditis elegans*, T6PP is essential for survival due to the toxic effect(s) of the accumulation of trehalose 6-phosphate. T6PP has also been shown to be essential in *Mycobacterium tuberculosis*. We determined the X-ray crystal structure of T6PP from *B. malayi*. The protein structure revealed a stabilizing N-terminal MIT-like domain and a catalytic C-terminal C2B-type HAD phosphatase fold. Structure-guided mutagenesis, combined with kinetic analyses using a designed competitive inhibitor, trehalose 6-sulfate, identified five residues important for binding and catalysis. This structure-function analysis along with computational mapping provided the basis for the proposed model of the T6PP-trehalose 6-phosphate complex. The model indicates a substrate-binding mode wherein shape complementarity and van der Waals interactions drive recognition. The mode of binding is in sharp contrast to the homolog sucrose-6-phosphate phosphatase where extensive hydrogen-bond interactions are made to the substrate. Together these results suggest that high-affinity inhibitors will be bi-dentate, taking advantage of substrate-like binding to the phosphoryl-binding pocket while simultaneously utilizing non-native binding to the trehalose pocket. The conservation of the key residues that enforce the shape of the substrate pocket in T6PP enzymes suggest that development of broad-range anthelmintic and antibacterial therapeutics employing this platform may be possible.

## Author Summary

Here, we describe the structure of trehalose-6-phosphate phosphatase (T6PP) from *Brugia malayi*. This enzyme is essential to the organism; deletion of the gene encoding T6PP results in toxic accumulation of trehalose 6-phosphate. Structure-guided mutagenesis coupled with kinetic analyses revealed residues important for binding and catalysis. The model for substrate binding suggests a binding mode in which shape complementarity plays a major role. Conservation of binding residues among T6PP orthologs present in pathogenic nematodes and bacteria favors T6PP as a suitable target for broad-range anthelmintic and antibacterial drug design.

## Introduction

Parasitic nematodes are responsible for devastating diseases that plague the tropical, low-income areas of Africa, Asia, and the Americas. In 2011, the World Health Organization estimated that 41% of the population worldwide was affected by these organisms [1]. Among the diseases caused by parasitic nematodes is the mosquito-transmitted lymphatic filariasis. It is projected that as many as 120 million people suffer from this disease with nearly 1.2 billion people at risk [2]. Lymphatic filariasis occurs upon infection of the lymphatic system by *Wuchereria bancrofti*, *Brugia malayi*, or *Brugia timori* and clinically manifests as lymphedema, hydrocele, and in the most extreme cases, elephantiasis. Related filarial nematodes inhabit other tissues; such infections may result in equally severe diseases, as exemplified by onchocerciasis or “river blindness” caused by *Onchocerca volvulus* infection. Filarial infections are responsible for extreme infirmity, distress, and social stigma. In fact, lymphatic filariasis is a major cause of permanent and long-term disability in people worldwide [3].

Due to the nature of infection and the impact on people suffering from this disease, the Global Program for the Elimination of Lymphatic Filariasis (GPELF) was established in 1999 with the major objective of ending the transmission of the disease by 2020. Currently the recommended regimen for treatment is the administration of albendazole together with either ivermectin (where onchocerciasis is endemic) or diethylcarbamazine citrate (where onchocerciasis is not present). Though community-wide treatment programs utilizing albendazole, ivermectin and/or diethylcarbamazine citrate have been effective, they are not without drawbacks. Side effects of albendazole and ivermectin are common, and although less frequent, they have also been observed with diethylcarbamazine citrate. Furthermore, these drugs only kill microfilariae, leaving the adult worms intact. Consequently, the drugs must be administered for the entire reproductive life span of the adult worm (approximately 5 years) [4]. In addition, the drug combination administered must be tailored to the specific parasite population in a given area because ivermectin administration can lead to encephalopathy in individuals with high microfilarial loads caused by *Loa loa* [5]. The development of new drugs that are not subject to these limitations is needed, particularly in the face of increasing drug resistance [6]. Indeed, drug resistance in veterinary nematodes is already widespread [7]–[9] and there are indications that in Ghana *O. volvulus* has developed a resistance to ivermectin [10].

To facilitate drug-discovery programs, the sequence determination of genomes of parasitic nematodes having human, domestic animal or plant hosts has been initiated [11]–[18]. The complicated life cycle of nematodes increases the difficulty of laboratory-based investigation. In fact, culturing *W. bancrofti* for *in vivo* studies has to date been unsuccessful. Fortunately, *B. malayi* can be maintained in a jird host [19] and is amenable to *in vitro* studies at different stages of its life-cycle [20]. Consequently, *B. malayi* now serves as a plausible model for research on lymphatic filarial nematodes alongside C. *elegans*, which for decades, has served as a model system for research on free-living nematodes. The similarities between *C. elegans* and parasitic nematodes with regard to genome sequences, and the phenotypes resulting from RNAi gene knockdown [21]–[23], indicate that *C. elegans*, being by far the most easily maintained organism, is an important resource in the development of broad-spectrum anthelminthic drugs.

Recently, top drug target candidates were identified in *B. malayi* using a ranking system [24], and among the highest-ranking targets is trehalose-6-phosphate phosphatase (T6PP) (UniProt: A8NS89), an enzyme that is required for the synthesis of trehalose [25]. T6PP is present in bacteria, fungi, plants, and invertebrate animals, but not in mammals. Trehalose is used by these organisms as an energy reserve, and it can also protect against environmental insults such as oxidative and osmotic stress, anoxia, heat, cold, freezing, desiccation, and anhydrobiosis [26], [27].

**Figure 1:**
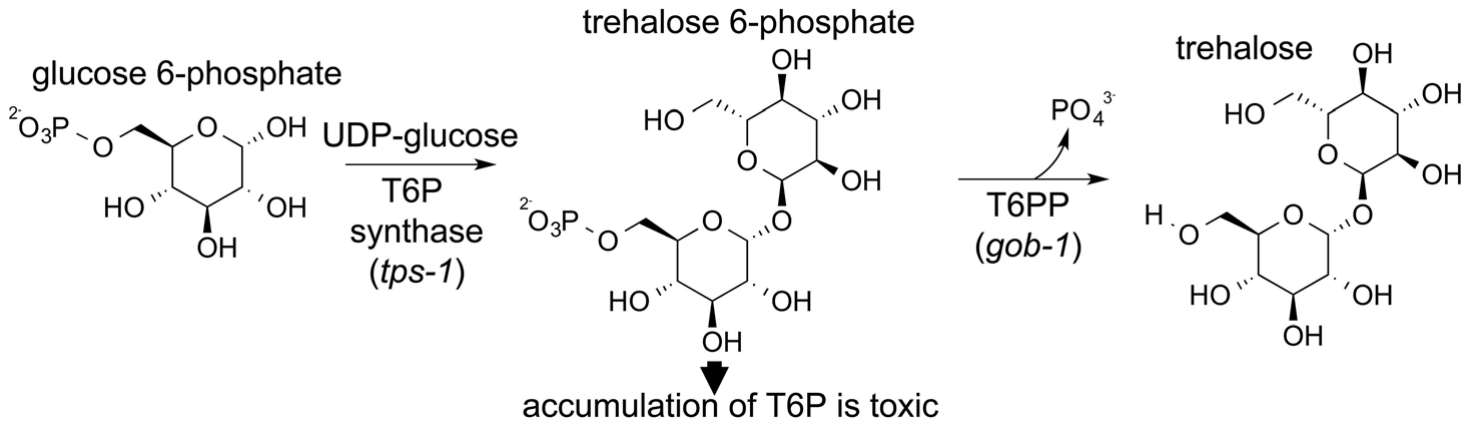
Schematic showing the two-step synthesis of trehalose. Trehalose is made in a two-step process catalyzed by trehalose-6-phosphate synthase (TPS) and trehalose-6-phosphate phosphatase (T6PP).

Trehalose is synthesized by a two-step pathway that involves T6PP and trehalose-6-phosphate synthase (genes *gob-1*, *tps-1* and *tps-2* in *C. elegans*, Figure 1). The synthase catalyzes the condensation of glucose 6-phosphate and UDP-glucose, forming trehalose 6-phosphate, and T6PP catalyzes phosphoryl transfer to water, forming trehalose. RNAi knockdown of the *C. elegans* T6PP-encoding gene *gob-1* (gut-obstructed 1) gives rise to larval lethality due to intestinal blockage and subsequent starvation [28]. Importantly, this phenotype is reversed by RNAi knockdown of the *tps-1* gene, suggesting that the lethality is due to a toxic accumulation of trehalose 6-phosphate [28]. A T6PP inhibitor might therefore bring about the same result, and thus we have targeted nematode T6PP for the development of small molecule anthelmintics.

As a first step toward inhibitor design the structure determination of T6PP was undertaken. The crystal structure of a putative T6PP has been reported from *Thermoplasma acidophilum* (PDB: 1U02) (29). Although this ortholog has low sequence identity, its structure identified it as a HAD superfamily (HADSF) phosphatase. All HADSF phosphatases possess a conserved Rossmann-fold catalytic domain, which contains the catalytic residues and the Mg^2+^ cofactor binding residues that together, constitute the substrate phosphoryl-group binding site. Most HADSF phosphatases, including the T6PP, also possess a cap domain (designated type C0, C1, C2A or C2B). During catalytic turnover the cap domain moves over the catalytic site through binding interactions with the substrate-leaving group, thereby forming an encapsulated active site. The size, shape and electrostatic properties of the active site are unique to each individual phosphatase. Although the sequences of the nematode T6P phosphatases are quite similar to one another, they share little identity with the *T. acidophilum* T6PP (12.7%). Moreover, sequence alignments revealed that the nematode orthologs possess a ∼140 amino-acid segment preceding the predicted N-terminus of the HAD phosphatase fold that is long enough to constitute a structural domain. It was thus both out of necessity for inhibitor design, and the intrigue for discovery of the fold and function of the novel N-terminal domain, that we pursued structure determination of the T6PP from *B. malayi*.

Herein, we report the X-ray structure of *B. malayi* T6PP and the findings from structure-activity analysis aimed at elucidating the role of the N-terminal domain and the active-site residues in substrate binding and catalysis.

## Results and Discussion

### Overall structure

**Figure 2:**
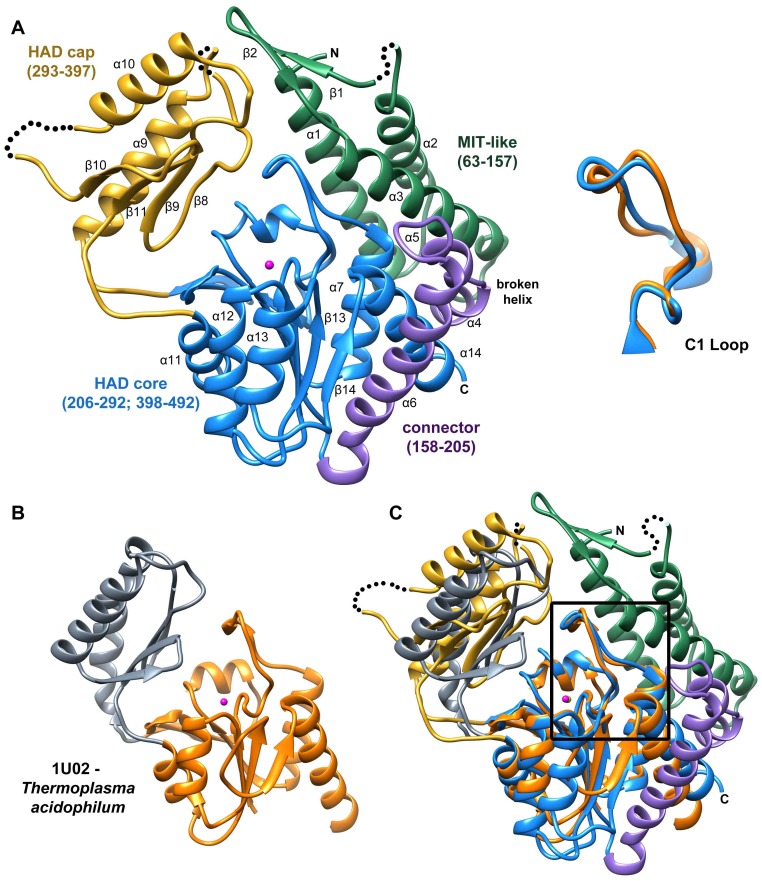
Structure of *B. malayi* T6PP and ortholog. Ribbon diagram of the X-ray crystal structure of T6PP from *Brugia malayi* with selected helices or strands labeled (PDB ID 4OFZ) with the MIT-like domain (green), connector region (purple) the catalytic core Rossmann-fold HAD domain (blue) and the HAD cap domain (gold) colored differentially (A). A single magnesium ion (magenta sphere) marks the active site. The *T. acidophilum* T6PP-like enzyme lacks the MIT-like domain found in *B. malayi* (B). An overlay of these enzymes reveals a slightly more closed cap orientation in the *T. acidophylum* enzyme, but a nearly identical conformation of the C1 loop (rmsd = 1.04 Å mainchain atoms) (C and above). This molecular figure and all others, unless otherwise noted, were generated with UCSF Chimera v1.8.

The structure of *B. malayi* T6PP bound to Mg^2+^ was determined at 3.0 Å resolution (residues 63–491, Figure 2A, Figures S1 and S2). Electron density was not observed for residues 1–62 and SDS-PAGE analysis of dissolved crystals revealed that this region was not lost during crystallization. These residues are not conserved among the nematode T6PP enzymes, and in the case of several *Caenorhabditis* species, they are absent (Figure S3). In addition, the *B. malayi* T6PP truncation mutant Δ59 (residues 1–59 deleted) retains the native fold (Figure S4) and the same catalytic activity as the wild type enzyme (Table 1). Thus, we conclude that residues 1–62 are probably disordered and are not required for stability or catalytic activity.

**Table 1:** Steady-state kinetic constants and dissociation constants for inhibitors for wild-type and variant *B. malayi* T6PP-catalyzed hydrolysis of trehalose 6-phosphate.

Construct	k_cat_ (s^−1^)	K_m_ (µM)	k_cat_/K_m_ (M^−1^s^−1^)	K_I_, trehalose 6-sulfate (µM)
wild type	24±2	360±60	6.9×10^4^	82±7
**Δ**59	28±1	260±20	1.1×10^5^	-
*Catalytic residues*				
D213A	NA^a^	-	-	-
D215A	∼0.0004	-	-	-
*Core residues*				
Y221A	1±0.05	330±50	3.0×10^3^	1,300±100
Y225A	6±0.1	150±10	3.6×10^4^	400±40
W280A	19±2	150±30	1.2×10^5^	270±50
N228A	18±0.8	40±8	4.5×10^5^	16±2
*Cap residues*				
Q332A	0.22±0.001	200±40	5.5×10^2^	1,000±200
R337A	0.28±0.03	400±100	1.5×10^3^	700±100
D378A	11±0.6	270±30	3.9×10^4^	320±30
S329A	4±0.2	250±50	1.5×10^4^	240±30
E384A	3±0.2	100±20	3.0×10^4^	200±30
K334A	0.06±0.007	500±120	1.1×10^2^	150±20
E386A	2±0.1	82±15	2.8×10^4^	150±20
T339A	15±1.0	200±40	7.7×10^4^	35±5
D336A	0.094±0.004	46±6	2.0×10^2^	33±5

a NA represents no activity detected above detection limit.

The overall T6PP structure consists of a three-helix bundle N-terminal domain (residues 63–157) connected via a short linker (residues 158–205) to the Rossmann-fold catalytic core domain (residues 206–292; 398–492) and the α,β-fold cap domain (residues 293–397), which together comprise the HADSF phosphatase structure (Figure 2A). Six parallel β-strands flanked by five α-helices form the core domain. A short β-hairpin (residues 282–292) precedes the cap domain (residues 293–397) and extends the central six-stranded β-sheet of the Rossmann-fold by two strands. The C2B-type cap domain is formed by an anti-parallel β-sheet topped by two α-helices (Figure 2A). Two short loops in the cap (residues 320–322; 367–370) were not well ordered and were not modeled.

The closest related structure is the T6PP-like enzyme from *T. acidophilum* (which does not possess the N-terminal domain (residues 1–205; Figure 2B) [29]. Superposition of the *B. malayi* and *T. acidophilum* structures (Figure 2C) gave a three-dimensional alignment with an overall rmsd of 2.6 Å compared to the rmsd of 2.1 Å obtained for the overlay of the individual core domains (14% sequence identity) and 2.2 Å rmsd for the overlay of the cap domains (9% sequence identity). Although the tertiary structures of the paired domains are nearly identical, the difference in global conformation resulting from distinct cap-core domain orientations observed for the two crystalline states, prevented structure determination by molecular replacement, thus necessitating phasing by single wavelength anomalous diffraction of the selenomethionine-substituted *B. malayi* T6PP.

### The MIT-like domain of T6PP

**Figure 3:**
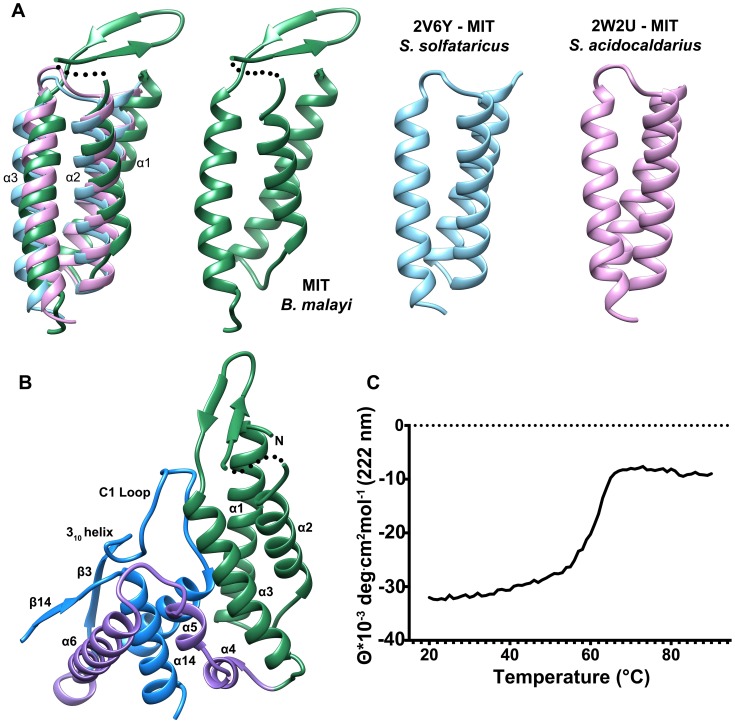
Comparison of the N-terminal MIT-like domain of T6PP with other MIT domains and its interaction with the HAD domain. The PDBeFold Server was used to identify structures with similar folds to that of the MIT-like domain. An overlay is depicted (A) between the MIT-domains from *B. malayi* T6PP (green) and the AAA ATPases Vps4 from *Sulfolobus solfataricus* (blue; PDB ID 2V6Y) and *Sulfolobus acidocaldarius* (pink; PDB ID 2W2U). The interaction between the α1–α3 interface of the MIT-like domain and the C1-loop of the HAD core domain is highlighted (B). Thermal stability analysis of *B. malayi* Δ59-T6PP reveals a strong interaction between the MIT and HAD domains. Thermal melt analysis by CD reveals one transition (C), suggesting the two domains are co-dependent.

The N-terminal domain (residues 63–157) consists of a three-helix bundle which is similar in topology to the Microtubule Interacting and Transport (MIT) domains of the Vps4-like ATPases from *Sulfolobus acidocaldarius* (PDB ID: 2W2U, rmsd 2.4 Å, Figure 3A) and *Sulfolobus solfataricus* (PDB ID: 2V6Y, rmsd 2.4 Å) [30], [31]. The MIT-like domain is tethered to the core domain by a helical-connector (residues 158–206) that is comprised of a broken α-helix (helices 4 and 5), which interacts with both domains and a second, longer α-helix (helix 6), which selectively interacts with the core domain (Figures 2A,3B). The presence of the MIT-like domain is striking as no other HADSF members are known to incorporate this domain. Sequence alignment analysis revealed that that the MIT-like domain is unique to the T6PP of nematodes and in organisms from the genus *Mycobacterium* (Figure S5 and S6).

MIT domains are protein-interacting domains typically associated with multivesicular body formation, cytokinetic abscission, or viral budding [32]. The best-characterized MIT domains are found in the essential AAA-ATPase Vps4. Vps4 is vital for endosomal trafficking to lysosomes, where it acts to dissociate ESCRT (endosomal sorting complexes required for transport) from membranes [30]. The MIT domain of Vps4 recognizes a conserved sequence in ESCRT-III termed the MIT-interacting motif (MIM). This sequence forms a protein-protein interaction site between the second and third α-helices of the Vps4 MIT-domain. However, MIT domains can interact with MIM motifs at each helical interface in the three-helix bundle, and can interact with multiple MIM motifs at once [33]. In T6PP, an intramolecular interaction occurs at the interface between the first and third α-helices of the MIT-like domain and the C1-loop of the HAD core domain (Figure 3B), leaving the solvent-exposed interface between the second and third helices free to make intermolecular interactions with other proteins. Although the protein-protein interface is the same as that seen in other MIM-MIT domain interfaces [33] the sequence of the C1-loop of T6PP does not share significant identity with any known MIM motif. The availability of a potential protein-protein interaction interface on the MIT-like domain raised the question of whether T6PP has a “moonlighting” [34] function in addition to or in conjunction with its enzymatic phosphatase activity.

The T6PP gene was originally identified in *C. elegans* and was named *gob-1* owing to the phenotype (gut obstruction) that occurs when the gene is knocked down. Knockdown of *gob-1* as well as the upstream T6P synthase genes *tps-1* and *tps-2* results in a normal phenotype [28]. This suggests that the *gob-1* lethality results from the buildup of the intermediate trehalose 6-phosphate, rather than the absence of trehalose (Figure 1). The presence of the MIT-like domain in the structure of T6PP led us to carry out further investigation. To test if the phenotype results from the absence of a protein-protein interaction between the T6PP MIT-like domain and another unknown protein, we repeated the RNAi experiments. Studies were carried out with the RNAi hypersensitive strain of *C. elegans eri-1(mg366); lin-15B (n744)*. We observed that feeding dsRNA of the T6PP *gob-1* gene results in arrest at the early larvae stage. In contrast, RNAi-hypersensitive worms which lack the T6P synthase gene *tps-1* and which are fed *gob-1* dsRNA showed a wild-type phenotype (Table S1). This observation is consistent with the earlier finding in wild-type *C. elegans* that the accumulation of T6P, rather than the lack of trehalose, is likely responsible for the observed lethality [28]. These results reinforce the suggestion that worm death is due to a metabolic effect, and that the gut obstruction phenotype is secondary to the accumulation of trehalose 6-phosphate. Moreover, the gene knock down lethality cannot be attributed to the removal of a hypothetical protein-protein interaction (through the MIT domain) because it occurs only if T6P synthase is present.

Deletion or mutation of the MIT-like domain is highly destabilizing to the enzyme and attempts to express the protein with the domain deleted (Δ179) or with the domain deleted and the potential hydrophobic patch “repaired” with the corresponding residues from the *T. acidophilum* T6PP (Δ179+L229Y/V232S/V236S/A243K) resulted in an unstable, insoluble form of the enzyme. The MIT domain itself was soluble and stable (T6PP:1-178-MIT Figure S7D). Notably, the loop region (C1-loop) that flanks the conserved active site forms extensive contacts with the MIT-like domain in the *B. malayi* protein, but the same C1-loop conformation is retained in the *T. acidophilum* ortholog lacking the MIT-like domain (Figure 2B). Thus, the interaction with the MIT-like domain is not necessary to retain the C1-loop conformation, although it is possible that in MIT-domain containing orthologs there is interdependence between the two structural units. Thermal unfolding observed by CD revealed one transition, suggesting that both domains unfold concurrently (Figure 3C). Additional proteolytic analysis confirms this observation (Figure S7) Together these findings suggest that the MIT-like domain and core domain are structurally co-dependent, and that any attempt to remove this interaction is detrimental to the overall stability of the protein. At this point it is uncertain whether the MIT-like domain plays a role in binding and catalysis of trehalose 6-phosphate hydrolysis.

### The active site of T6PP

The active site of HADSF members lies at the interface of the cap and core domains, with the core domain comprising the phosphoryl-transfer catalytic machinery. Indeed, the *B. malayi* T6PP core holds the four conserved active-site motifs that, in the HADSF [35], coordinate both the magnesium cofactor and phosphoryl group of substrate for catalysis. A single magnesium ion is observed to be coordinated in the active site by two waters and four residues (Motif I: Asp213, Asp215 (mainchain C = O oxygen), Motif IV: Asp424 and Asp428). Thr253 (Motif II) and Lys398 (Motif III) presumably form a hydrogen bond with the phosphate during catalysis.

In *B. malayi* T6PP, the cap was found to be in an open orientation relative to the core, though the crystal contacts were consistent with stabilization of the protein in this conformation and the protein may exist in alternate conformations in solution. Notably, the *B. malayi* T6PP has a slightly more open conformation than the orthologous *T. acidophilum* enzyme (Figure 2C). Because the cap domain mediates interactions with the substrate leaving group, but only the closed conformation positions substrate-binding residues for binding and catalysis, initial analysis of the structure did not reveal obvious residues involved in substrate recognition. Moreover, attempts to obtain a liganded structure or closed conformer structure have been unsuccessful thus far.

**Figure 4:**
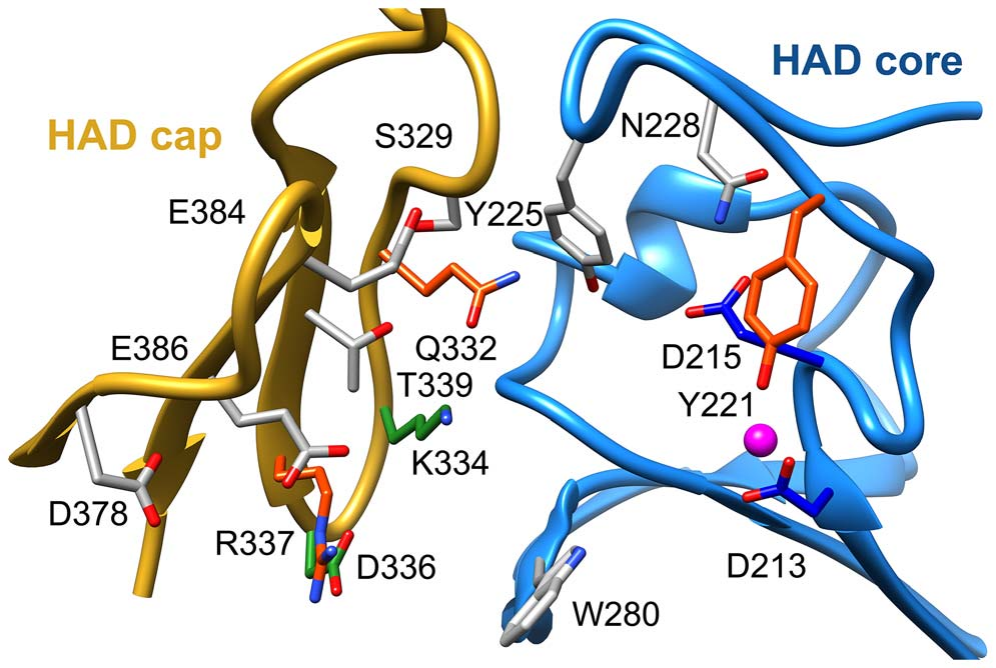
Putative substrate interacting residues in *B. malayi* analyzed by mutagenesis and kinetics. Residues in the cap and core regions analyzed by mutagenesis are depicted as sticks and colored according to kinetic parameters: no effect on kinetic parameters (grey); catalytically inactive (blue); decreases in k_cat_ (green); significant changes in k_cat_ and K_I_ for a T6S substrate analog (red).

To identify residues important for ligand binding, structure-guided single site mutagenesis coupled with enzyme kinetics was performed. Several residues near the active site or in the cap region were selected for mutagenesis (Table 1, Figure 4). These residues are present on the face of the β-sheet that is oriented toward the active site and are highly conserved among T6PP enzymes from nematodes, but were not as conserved among T6PP enzymes from other phyla including *Mycobacterium*, other prokaryotes and *Saccharomyces* (Figure S6). As expected, replacement of the catalytic Asp213 or Asp215 resulted in the loss of all detectable activity (Table 1). None of the other replacements resulted in a dramatic change in K_m_ for T6P. However, mutations in Tyr221, Gln332, Lys334, Asp336, or Arg337 resulted in large decreases in k_cat_ (24-, 110-, 400-, 255- or 85-fold, respectively) suggesting a role in catalysis. Each of these residues except for Tyr221 are found in the cap domain (Figure 4) and may interact with the sugar moiety in order to orient the substrate for catalysis. Since the observed changes in k_cat_/K_m_ stemmed from changes in k_cat_, we moved to inhibitor binding studies to test if any of the protein variants differed in their affinity for substrate.

To assess substrate affinity, the substrate analogue trehalose 6-sulfate (Materials and Methods) was used to measure K_I_ for all of the T6PP variants (Table 1). Trehalose 6-sulfate was shown by steady-state inhibition kinetics to be a competitive inhibitor of wild type T6PP with a K_I_ of 82±7 µM against trehalose 6-phosphate as substrate. Three of the variants that showed a decrease in k_cat_ also showed dramatic increases in K_I_ for trehalose 6-sulfate: Tyr221, Gln332 and Arg337 (Table 1, Figure 4). From these experiments, Tyr 221, Gln332, and Arg337 are likely to be involved in binding trehalose 6-phosphate, while Lys334 and Asp336 may play other roles in catalysis. These roles may include the exclusion of solvent from the active site, steric restraint of the substrate for catalysis, or the positioning of other residues required for enzyme activity.

To determine whether homologous residues could play a role in T6PP orthologs, homology models of the enzymes from *E. coli*, *S. cerevisiae*, and *M. tuberculosis* were generated [36] and compared to the structure from *B. malayi* and *T. acidophylum* (because sequence identity was low, a homology model which utilizes additional constraints was the most reliable way to ensure correct alignment). The residues that affected both catalysis and K_I_ were found to be conservatively replaced in each of these orthologs (the bulky residue Tyr221 is replaced by Ile; Gln332 is replaced by Glu/Tyr; Arg337 is replaced by Lys) (Figure S8), reinforcing the importance of the roles of the residues identified here. The presence of these residues in all T6PP enzymes examined suggests that design of broad-spectrum inhibitors may be possible.

### Mutagenesis-guided model of the T6PP-trehalose 6-phosphate complex

**Figure 5:**
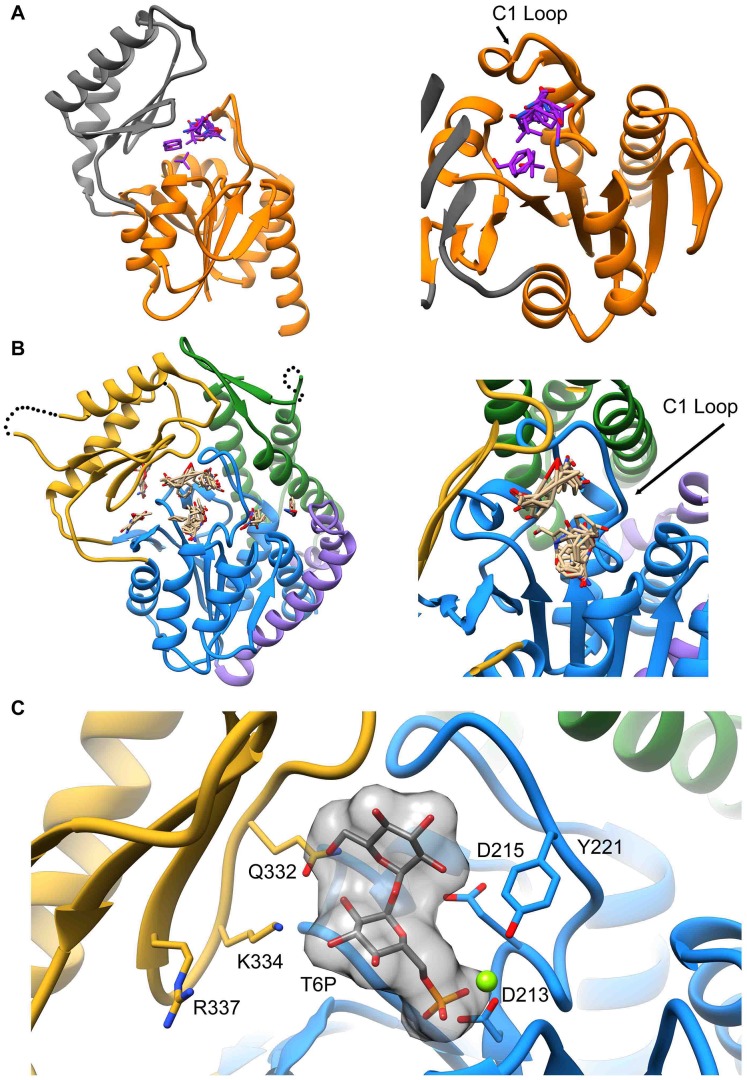
Proposed model of trehalose 6-phosphate in the active site of T6PP. The FTMap server was used to identify hot spots where protein-substrate interactions may occur. Analysis of the T6PP enzyme from *T. acidophilium* (1U02) (A), and *B.* malayi (B) reveal hot spots near the interface of the cap and core domains. These hot spots are cradled by the structurally conserved C1-Loop. T6P was placed manually into the active site of T6PP by coordinating the Mg^2+^ cation with the phosphate group (C). The residues identified as important via mutagenesis and kinetics are labeled and can be seen in proximity to the trehalose moiety.

Attempts to visualize the closed-cap form of the *B. malayi* enzyme using molecular dynamics simulations were unsuccessful (*see* *Materials and Methods*). To provide a platform for inhibitor design, a model of T6PP with trehalose 6-phosphate bound in the active site was constructed. To accomplish this, trehalose 6-phosphate was manually placed in the active site of T6PP such that it was positioned for “in-line” attack by Asp213 (the Asp nucleophile) and coordinated to the Mg^2+^ cation as has been observed in all HADSF enzyme-substrate or enzyme transition-state analogue complex structures [37]–[40]. In addition, the side chain of Asp215 (the general acid/base catalyst conserved in phosphatase members of the HAD superfamily [35]) was positioned to promote leaving-group protonation. To position the trehalose moiety, the FTMap server [41], [42] was used to identify putative hot spots in the cap and core domains of both the *B. malayi* and *T. acidophilum* T6PPs. Intriguingly, FTMap analysis of the two enzymes revealed several hot spots forming a pocket extending from the phosphate-binding site to the C1 loop (Figures 5A,B). The identified binding pocket was juxtaposed to the important residues identified by mutagenesis in *B. malayi* in the cap (Figure 5B). The trehalose moiety was manually rotated to fit within the hot-spot binding sites and the model was then minimized using NAMD [43]. Tyr221, Lys 334, Gln332 and Arg337 are positioned near the substrate and, consistent with the mutagenesis results, may play important roles in binding (Figure 5C). Overall, the interface between the cap and core domains forms a substrate-binding pocket for trehalose 6-phosphate that may exclude solvent, provide steric constraint through van der Waals contacts, and may form only a few specific hydrogen bonds.

Although the T6P docked in the crystal structure of *B. malayi* T6PP brings the proposed binding residues proximal to the substrate, the model is not in a closed conformation. FTMap analysis suggests that the *T. acidophilum* T6PP enzyme is in a more closed conformation compared to that of *B. malayi*. Superimposition of the *B. malayi* cap with the *T. acidophilum* T6PP cap and subsequent analysis by DynDom [44]–[46] predicts that the cap rotates 45.6° with respect to the core (Figure S9A). Placement of the *B. malayi* cap in this orientation positions the residues identified by mutagenesis within contact distance of the predicted T6P model position (Figure S9B).

### Comparison to other HAD phosphatases

**Figure 6:**
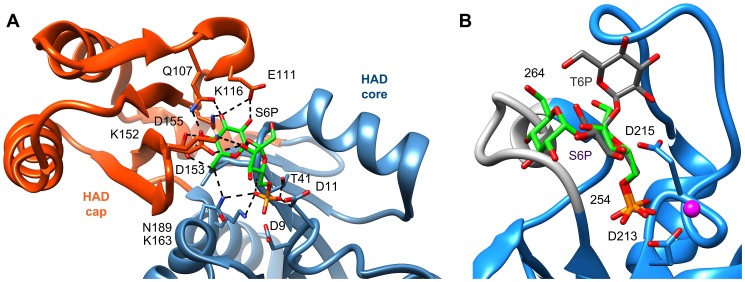
Comparison of ligand binding between sucrose-6-phosphate phosphatase and trehalose-6-phosphate phosphatase. An analysis of the crystal structure of the S6PP-sucrose 6-phosphate (S6P) complex (PDB: 1U2T) and the model of the T6PP-trehalose 6-phosphate (T6P) complex revealed that the stereochemistry of the glycosidic bond might affect specificity. The cap can be found in a different position in the S6PP-S6P complex than either the crystal structure or cap-closed model of T6PP, affecting the size and shape of the active site cavity. An extensive hydrogen-bonding network exists between S6PP and S6P (black dashed lines), utilizing residues from both the cap domain (orange) and the core domain (blue) (A). Positioning of the cap in S6P may be impacted by the α(1→2)β glycosidic bond of sucrose 6-phosphate versus the α(1→1)α glycosidic bond in trehalose 6-phosphate. An overlay of T6P from our model and S6P reveals a putative clash between S6P and residues 254–265 (light grey loop) in T6PP (B) potentially explaining the lack of turnover or binding of S6P.

To compare our model of the T6PP-T6P complex to other phosphatases the DALI server [47] was used to identify HAD members with similar structures. Because the orientation between cap and core varies and the cap is the most variable portion of HAD family members, the cap domain from T6PP was used to find similar structures with C2B-type caps. Besides the T6PP-like enzyme from *T. acidophilum*, one of the highest scoring structures was that of sucrose-6-phosphate phosphatase (S6PP) from the cyanobacteria *Synechocystis sp.* PCC 6803 [48]. Sucrose plays a similar role in cells to that of trehalose, and is synthesized in response to osmotic stress in a two-step pathway involving the dephosphorylation of a sucrose 6-phosphate (S6P) intermediate [49], [50]. The structure of the S6PP-S6P complex (PDB: 1U2T) reveals a closed cap conformation with an extensive hydrogen-bonding network providing substrate stabilization (Figure 6A). The ability of these enzymes to discriminate between T6P and S6P may be due to the different glycosidic-linkages of sucrose and trehalose (sucrose: α(1→2)β vs. trehalose: α(1→1)α). According to our model, T6P packs against Tyr221 in the C1-Loop and may be stabilized for catalysis via hydrogen bonds from Gln332 and Arg337. S6P is oriented in the opposite direction and is stabilized by several hydrogen bonds from cap-residues (Figure 6A). The C1-loop in S6PP is much shorter and less pronounced, emphasizing its importance in T6P recognition by T6PP.

**Figure 7:**
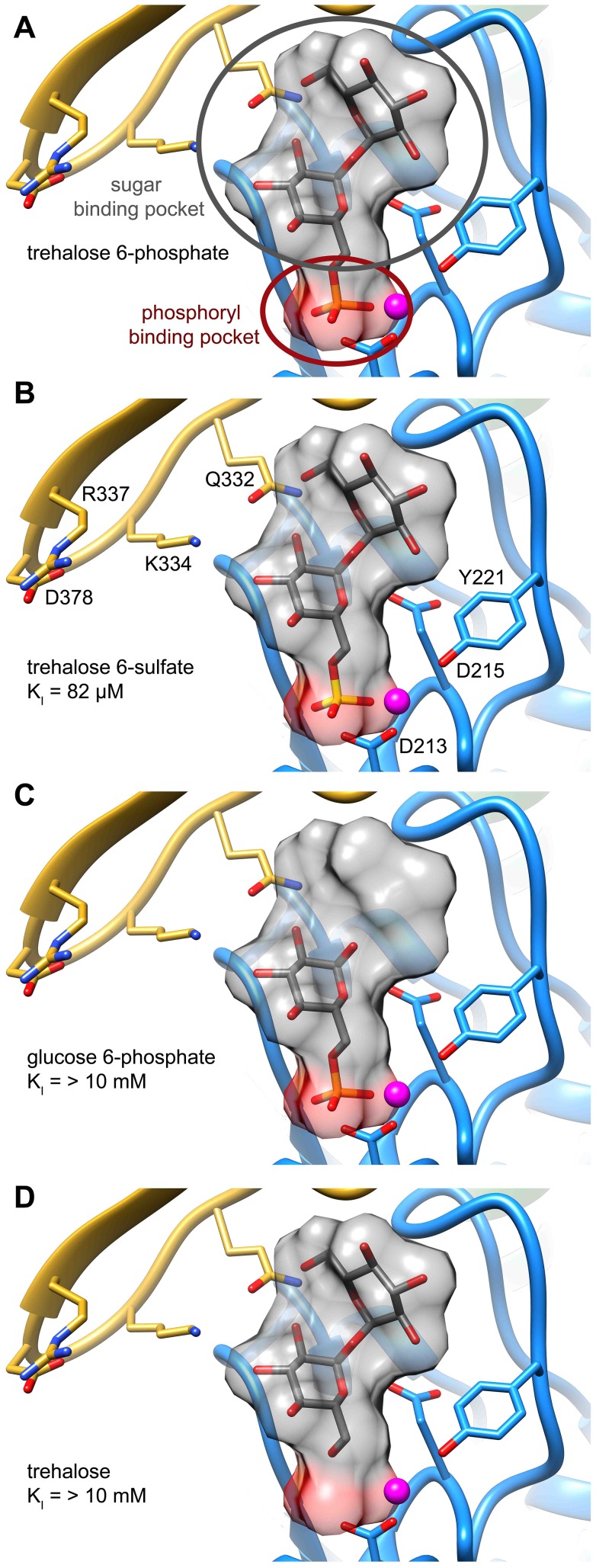
Two binding pockets in the T6PP enzyme. Analysis of the hypothesized binding model for trehalose 6-phosphate and enzyme kinetics suggests that inhibitors should interact with two pockets in order to maximize interactions. Trehalose 6-phosphate (A) and trehalose 6-sulfate (B) presumably bind in the phosphoryl-binding and sugar-binding pockets while glucose 6-phosphate (C) and trehalose (D) interact in only one pocket.

Overlay of S6P in the active site of T6PP reveals a steric clash with a loop in T6PP (Figure 6B). This is consistent with the fact that T6PP does not accept S6P as a substrate as well as the inability of S6P to bind to or act as an inhibitor of T6PP. In addition, the use of hydrogen bonds as a mode of sugar recognition in S6PP versus van der Waals interactions in T6PP explains the inhibition of S6PP by the monosaccharide glucose moiety comparable to that of sucrose itself [48]. In contrast in T6PP, we found that there is no inhibition by the trehalose or glucose 6-phosphate substituents of trehalose 6-phosphate (K_I_>10 mM for these compounds, Figures 7C, D). Thus, it is expected that a critical issue in designing inhibitory ligands for T6PP will be the occupancy of the phosphoryl binding site in conjunction with shape recognition of the leaving group (Figures 7A, B). Indeed, T6PP acts as an excellent system for the exploration of inhibitory ligands of phosphoryl binding sites as the majority of inhibitors to phosphoryl transfer enzymes [51] take advantage of adjacent binding sub-sites (e.g. the nucleotide or peptide/protein binding subsites in kinases [52], [53] rather than the transferring phosphate site.

### Concluding remarks

In order to improve our understanding of the structure/function relationship in T6PP enzymes from parasitic nematodes we determined the structure of T6PP from *B. malayi*. Although the cap-open, unliganded form was observed in the structure, site-directed mutagenesis coupled with substrate and inhibition kinetics identified five residues (Table 1, Figure 4) important for catalysis and/or substrate affinity. Notably, no single residue is critical to binding, consistent with a mode of binding of the trehalose moiety by van der Waals contacts and shape complementarity between the sugar and enzyme. Moreover, the trehalose binding site is not dominated by hydrophobic residues that could form ring-stacking interactions like those found in other carbohydrate binding proteins [54]–[57]. The conservation of several key binding site residues among T6P phosphatases from parasitic nematodes and pathogenic bacteria indicates that a common strategy for T6PP inhibitor design might be used in the development of antibiotics as well as anthelmintics.

An inhibitor design strategy is now afforded by the availability of the T6PP structure. Based on inspection of the structure and the inhibition kinetic analysis of wild-type and mutant *B. malayi* T6PP, there are two independent regions that can be simultaneously targeted for inhibitor design, namely the phosphoryl group binding site and the trehalose leaving group binding site (Figure 7). The lack of inhibition of the *B. malayi* enzyme by trehalose (K_I_>10 mM) compared to the observed tight binding of the inert substrate analog trehalose 6-sulfate (K_I_ = 82 µM) shows that electrostatic interaction with the phosphoryl group binding site provides a significant amount of binding energy that can be captured by a strategically positioned inhibitor sulfate group. Although the T6P trehalose moiety is essential for properly orienting the substrate in the catalytic site for catalytic turnover, it does not appear to provide the amount of binding energy needed for a lead inhibitor. Thus, we envision the design of a bi-dentate inhibitor comprised of the high affinity sulfate group (or similar) for interaction with the catalytic site of the core domain with an organic moiety that maximizes the intrinsic binding energy derived from hydrophobic and electrostatic interaction with the binding region made available by the interfaced cap domain (not necessarily mimicking those made by the substrate trehalose moiety).

## Materials and Methods

### Bioinformatic analysis of the T6PP enzyme family

Sequences of T6PP enzymes were downloaded from UniProt, aligned using T-Coffee [58] and visualized using ESPript [59]. A sequence alignment of all T6PP enzymes from nematodes revealed a high degree of conservation with the exception of the N-termini which vary in length (1–60 amino acids) and sequence (Figure S3). In addition, an extra N-terminal domain consisting of approximately 150 residues was found in T6PP enzymes from nematodes and in organisms from the genus *Mycobacterium* (Figs. S5 and S6)

### Protein preparation and crystallization

The gene *Bm1_08695* encoding T6PP from *B. malayi* was cloned from a cDNA library by PCR using primers with the restriction sites for NdeI and BamHI embedded. Standard cloning procedures were used to place the gene into a modified pET-15b vector (thrombin site replaced with a TEV site; pET-15(TEV)). The individual domains were also separately cloned into a pETDUET-1 vector by placing the HAD core (180–492) into MCS1 using XbaI and BamHI and placing the MIT domain (1–179) into MCS2 using NdeI and KpnI. To create variants for probing residues important to activity (Table S2), the Q5 Site-Directed Mutagenesis Kit (New England Biolabs, NEB) was used following the manufacturer's protocol. NEB 10-beta Competent *E. coli* (NEB 3019) were used for plasmid propagation and purification, and all plasmids were sequenced to confirm accuracy before use. For expression, T6PP, T6PP-Δ59, T6PP-Δ179, T6PP:1-178 (MIT) or the T6PP variants were prepared with T7 Express *lysY/I^q^* Competent *E. coli* (NEB C3013) that were transformed with appropriate expression vectors. Flask cultures were grown in LB medium containing 0.1% glucose. Fermentation medium was as follows: 2% soytone, 1% yeast extract, 171 mM NaCl, 1.87 mM KH_2_PO_4_, 5.97 mM Na_2_HPO_4_, 13.1 mM NH_4_Cl, 0.5 mM K_2_SO_4_, 8% glycerol, 5 mM MgSO_4_, 10 mM betaine, 1× Korz trace metals [60], and 0.01% antifoam 204 (Sigma). For flask cultures 100 µg/ml of ampicillin was used and 150 µg/ml ampicillin was used in fermenters. One colony was inoculated into 7 ml of LB and 0.1 ml of the suspension was inoculated into 500 ml of LB for seed cultures. The seed cultures were incubated overnight at 30°C to an OD_600_ of 2. Ten liter Fermentations were done in 19.7 L Bioflo 510 fermenters (Eppendorf). The pH was controlled at 7.0 with automatic addition of 30% NH_4_OH and 46 N H_3_PO_4_. The dissolved oxygen was controlled at 20% of air saturation using constant gas flow at 0.5 vvm and an agitation-oxygen cascade. The culture was grown at 30°C to an OD_600_ of 4. IPTG was then added to 0.5 mM and the temperature was dropped to 18°C for induction. The culture was induced for 18 h before harvesting. Cells containing pET-15b (TEV):T6PP-Δ179 were induced with 1 mM IPTG for 3 hours at 37°C once the OD_600_ reached 0.6–0.8. Cells were pelleted and frozen at −20°C until further processing. For lysis, cell pellets were thawed and resuspended (3 ml buffer per g pellet) in a solution consisting of 50 mM Tris pH 7.5, 500 mM NaCl, 20 mM imidazole, 1 mM EDTA, 1 mM PMSF, 30 µg/ml DNase I (Sigma) and 1 protease inhibitor cocktail tablet (Sigma S8830) per every 100 g of cell pellet. Cells were lysed via a single pass through a microfluidizer (Microfluidics Model #M110P) at 20,000 PSI. Lysate was clarified at high speed (100,000× g), bound to a HisTrap FF column using an ÄKTA FPLC (GE Biosciences) and washed extensively before elution using a gradient with a buffer consisting of 50 mM Tris pH 7.5, 500 mM NaCl, 500 mM imidazole. The resulting protein fraction was then diluted 20-fold in a low-salt buffer consisting of 20 mM Tris pH 7.5 and 10 mM NaCl and bound to a HiTrap Q column (GE Biosciences). The protein was eluted using a gradient with a buffer comprised of 25 mM Tris pH 7.5 and 1 M NaCl. The N-terminal His-tag was removed by TEV protease (Blommel and Fox, 2007) using a 1∶50 ratio (mg∶mg) of TEV:T6PP prior to dialysis into a storage buffer consisting of 25 mM Tris pH 7.5, 10 mM NaCl, 5 mM MgCl_2_. Purified T6PP was concentrated to 15 mg/ml using an Amicon Ultra concentrator (10K MWCO, Millipore), aliquoted in small volumes and stored at −80°C.

To prepare selenomethionine-incorporated protein, the same procedure was used with two minor changes. Protein was expressed in the T7 Express Crystal Competent *E. coli* methionine-auxotroph cell strain (NEB C3022), following the manufacturer's protocol. Second, all buffers used contained 5 mM DTT to prevent oxidation of selenium.

Initial crystal screens were set up with either full-length T6PP or T6PP-Δ59 at 15 mg/ml in 96-well microplates (Corning 3552) using the Crystal Screen HT, PegRx HT and Index HT sparse matrix crystallization screens (Hampton Research) and a final drop size of 1.5 µl at 290 K. The full-length protein yielded hexagonal crystals in several low molecular weight polyethylene glycol (PEG) solutions buffered within a pH range of 5.0–8.5 and were optimized using the Additive HT and Detergent HT screens (Hampton Research). For data collection, optimized crystals of native T6PP were grown at 290 K in hanging drops (concentration of 15 mg/ml) in 33% PEG 300, 0.1 M sodium citrate pH 5.0, 13% ethylene glycol (EG) and 10 mM CoCl_2_. For cryoprotection, the concentration of EG was slowly increased to 25% by adding a concentrated solution directly to the crystal drop. Crystals were harvested using CryoLoops (Hampton Research), looped through LV CryoOil (MiTeGen), frozen in liquid nitrogen and stored until data collection. Crystals of selenomethionine-incorporated T6PP were grown at 290 K in hanging drops (concentration 10 mg/ml) in 30% PEG 300, 0.1 M sodium citrate pH 5.0 and 20 mM NDSB-256. For cryoprotection, 2-methyl-2,4-pentanediol (MPD) was slowly added to crystallization drops (final concentration of 25%). Crystals were harvested and stored in the same way as the native crystals.

### X-ray data collection and structure determination

Native and derivative datasets were collected at either the National Synchrotron Light Source (NSLS, beamline ×25) or the Stanford Synchrotron Radiation Lightsource (SSRL, beamlines 7-1, 12-2) using automounter systems. Data was collected under nitrogen gas flow at 100 K in 1° oscillations using either a Dectris Pilatus 6M detector (NSLS ×25; SSRL 12-2) or an ADSC Quantum 315r detector (SSRL 7-1) and processed with HKL-3000 [61]. Initial electron density maps were calculated via single-wavelength anomalous dispersion (SAD) with Phenix AutoSol using a 3.4 Å selenomethionine derivative dataset. This revealed 15 anomalous selenium peaks corresponding to 15 (out of 16) selenomethionine residues. Phases were improved and extended to 3.0 Å with Phenix AutoBuild using a native dataset [62]. The model was built iteratively with Coot [63] and refined in Phenix Refine [62] with 10% of the reflections excluded for the calculation of R-free. The final model converged to an R-value of 21.5% and an R-free of 25.9% with 93.2% of residues in the favored region and 6.6% of residues in the allowed region of the Ramachandran plot (Lovell et al., 2003). Full data collection and refinement statistics are summarized in Table S3.

The quality of the completed model was assessed by MolProbity [64]. Structural similarity to known folds was determined using either the PDBeFold (http://www.ebi.ac.uk/msd-srv/ssm) [65] or DALI (http://ekhidna.biocenter.helsinki.fi/dali_server/start) [47] servers.

### Generation of *tps-1* knockout *C. elegans* for RNAi studies

The *eri-1(mg366); lin-15B(n744) tps-1(ok373)* worms were constructed by crossing *tps-1(ok373)* worms with *eri-1(mg366); lin-15B(n744)* worms. The genes *lin-15B* and *tps-1* are closely linked on the X chromosome. Animals (n = 600) were genotyped to identify a recombination event that occurred between *lin-15B* and *tps-1*. Progeny (n = 600) of worms heterozygous for *tps-1(ok373)* and *lin-15B (n744)* were picked individually to a petri plate. After the worms generated progeny on the new plate the genomic DNA from each parent worm was isolated as previously described [66]. The genomic DNA of each worm was screened by PCR for homozygosity of the *tps-1(ok373)* deletion allele and heterozygosity of the *lin-15B (n744)* allele. Homozygosity for *tps-1(ok373)* was determined by the presence of the deletion mutation and absence of the wild-type gene. Additionally, the worms were tested for the presence of the *lin-15B (n744)* mutation by performing PCR of the *lin-15B* locus and digesting the product with BccI, which only cuts the mutant n744 mutant *lin-15B* allele. Only animals that were homozygous for the *tps-1(ok373)* deletion allele and heterozygous for *lin-15B(n744)* would had the recombination event between the two genes that was necessary for the *eri-1(mg366); lin-15B(n744) tps-1(ok373)* strain construction. Finally, the homozygosity for the *eri-1(mg366)* mutation was established by PCR and sequencing. L4 worms (n = 45) of each genotype were picked to individual plates with HT115 bacteria expressing *gob-1* dsRNA [67]. The progeny of each animal was examined for defects.

### Synthesis of trehalose 6-sulfate

To a solution of SO_3_/C_5_H_5_N complex (432 mg, 2.7 mmol) in freshly distilled pyridine (5 mL) was added a solution of the benzyl-protected trehalose (1.345 g, 1.35 mmol) in freshly distilled pyridine (5 mL) was added. The mixture was stirred for 5 h at room temperature, neutralized by addition of Na_2_CO_3_(aq) (1 M, 10 mL) and concentrated under reduced pressure. The residue was titrated with anhydrous methanol and the triturate was filtered. The filtrate was concentrated *in vacuo* giving a residue that was subjected to silica gel column chromatography (30∶1 MeOH/EtOAc) affording the protected trehalose 6-sulfate (1.35 g, 93%) as a white powder. A solution of the white powder (0.96 g, 0.89 mmol) in MeOH/H_2_O (1∶1,V/V, 40 mL), 20% Pd(OH)_2_/C (1.26 g) was repeatedly purged with hydrogen. The resulting mixture was stirred under 1 atm of H_2_ at room temperature for 24 h and filtered through a Celite pad. The filtrate was concentrated *in vacuo* giving a residue that was subjected to silica gel column chromatography (1∶4∶4, water/isopropanol/ethyl acetate) to give trehalose 6-sulfate as a white powder (366 mg, 93%). TLC (water/isopropanol/ethyl acetate, 1∶4∶4, v/v/v): Rf = 0.40; ^1^H NMR (D_2_O): 3.39–3.52 (2H, m), 3.60–3.67 (2H, m), 3.72–3.86 (5H, m), 3.97–4.0 2(1H, m), 4.23–4.26 (2H, m), 5.16–5.18 (2H, m); ^13^C NMR (D_2_O): 60.3, 66.7, 69.1, 69.5 70.0, 70.68, 70.74, 72.0, 72.19, 72.25; HRMS(ES) m/z: [M+Na+] calculated for C_12_H_21_O_14_S, 421.0652; found, 421.0648.

### Substrate specificity profile and steady-state kinetic characterization of T6PP

To determine the substrate specificity profile for T6PP, purified enzyme was diluted to 10 µM into a buffer consisting of 25 mM Tris pH 7.5, 50 mM NaCl, 5 mM MgCl_2_ and 1 mM DTT and aliquoted into 96-well plates in 5 µl volumes. Equivalent volumes of 20 mM substrate from a 167-compound in-house screen were then added and incubated for 45 minutes at room temperature. Next, 52.5 µl of BioMol Green (Enzo Life Sciences) was added to the wells to detect enzymatically released phosphate and incubated for another 30 minutes. After incubation, the absorbance of each well at 625 nm was measured using a Molecular Devices SpectraMax M5 microtiter plate reader. T6PP was found to be highly specific for trehalose 6-phosphate, eliminating the need to measure steady-state kinetic constants for other substrates.

For steady-state kinetic characterization, purified T6PP and the variants made by site-directed mutagenesis were diluted to between 25–75 nM into a buffer consisting of 25 mM Tris pH 7.5, 50 mM NaCl and 5 mM MgCl_2_. The steady-state kinetic parameters (K_m_ and k_cat_) for trehalose 6-phosphate were determined from initial reaction velocities measured at varying concentrations (0.0625–5 mM) using the EnzCheck Phosphate Assay Kit (Invitrogen). Absorbance measurements were performed with a Beckman DU800 spectrophotometer using quartz cuvettes (Starna Cells, 18B-Q-10) and 250 µl volumes. Data were fit to the following using SigmaPlot Enzyme Kinetics Module:
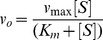
Here, v_o_ is the initial velocity, v_max_ the maximum velocity, [S] the substrate concentration and K_m_ the Michaelis-Menten constant calculated for trehalose 6-phosphate. The value for k_cat_ was calculated from the following:
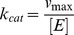
where [E] is the protein concentration in the assay. The steady-state kinetic constants for T6PP and its variants made via site-directed mutagenesis are reported in Table 1.

The steady-state competitive inhibition constant K_I_ was determined for trehalose 6-sulfate by fitting the initial velocity data, measured as a function of trehalose 6-phosphate (0.5 K_m_ to 5 K_m_) and inhibitor (0, K_I_, 2 K_I_) concentration to the following using SigmaPlot Enzyme Kinetics Module:
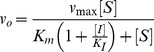
where [I] is the inhibitor concentration and K_I_ is the inhibition constant.

### Homology modeling of related T6PP enzymes

To generate homology models of related T6PP enzymes, FASTA sequences for *E. coli* (UniProt: E8Y507), *S. cerevisiae* (A6ZY39), and *M. tuberculosis* (H8EZ37) were uploaded to the Protein Homology/analogY Recognition Engine V 2.0 (Phyre2) server, where models were automatically generated using the best possible template model [36]. In this case, each model was based on 1U02 with a >95% confidence level. To model the putative MIT-like domain in *M. tuberculosis*, one-to-one threading on the Phyre2 server was used utilizing the structure of T6PP from *B. malayi* as the template.

### Hot spot mapping using the FTMap server

To map substrate binding residues in the HAD domain, the FTMap server (http://ftmap.bu.edu) was used to identify hot spots. To map putative MIT interactions, PDB files consisting of only the MIT domains (residues 63–156 for *B. malayi*; 2W2U chain A; 2V6Y chain A) were uploaded and analyzed in PPI mode. To map putative substrate binding residues, PDB files (residues 63–491 for *B. malayi*; 1U02 chain A) were uploaded and analyzed using default settings.

### Modeling of the T6PP-T6P complex

To model the T6PP-T6P complex, trehalose 6-phosphate was built (α-D-glucopyranosyl-(1→1)-α-D-glucopyranoside 6-phosphate) and manually placed in the active site of T6PP. The phosphate was placed such that it was positioned for “in-line” attack by Asp213 (the Asp nucleophile) and coordinated to the Mg^2+^ cation. In addition, Asp215 (Asp+2, general acid/base residue) was positioned to promote leaving-group protonation. The sugar was manually rotated to lie against the C1-loop guided by mutagenesis and FTMap hot spot results. The model was then minimized using NAMD [43] and analyzed using UCSF Chimera [68].

### Molecular dynamics simulations

Hydrogen atoms were added to the docked T6PP-T6P model, using the HBUILD function in CHARMM [69] and the coordinates gently minimized in general Born implicit solvent (GBIS) [70] by gradually removing harmonic restraints on the system heavy atoms and performing 25 steps of steepest descent followed by 250 to 500 steps of advanced basis Newton-Raphson (ABNR) steps. A final, unrestrained minimization using ABNR steps was performed until the energy gradient stabilized below 10^−5^ kcal/mol-step. A further 200 steps of minimization was performed in NAMD [43] and rigid bonds. GBIS solvation was employed with an effective ion concentration of 150 mM and a 12 Å cutoff for calculating the Born derivatives. All other parameters were set to NAMD defaults. Surface tension terms in the solvation model were ignored. A non-bonded cutoff distance of 15 Å was employed with a switching function employed to 16 Å. Langevin dynamics was initiated at 300 K utilizing a damping coefficient of 1/ps, a 2 fs timestep and rigid bonds enforced. This configuration was integrated for a total of 1.2 ns.

### Accession numbers

Coordinates for T6PP and structure factors have been deposited in the Protein Data Bank (PDB ID: 4OFZ).

## Supporting Information

Figure S1 **2F_o_-F_c_ electron density maps for Bm T6PP countered at 1.5σ.** Maps were calculated by manually omitting the core β-sheet of the Rossmann fold and carrying out a round of simulated annealing. Both panels show the same region in either ribbon form (above) or sticks (below).
(TIF)

Figure S2 **Topology model for Bm T6PP.** A modified topology diagram from the Topsan server (http://www.topsan.org) is depicted with the same coloring scheme as the structure in Figure 2.
(TIF)

Figure S3 **A variable N-terminus and conserved MIT-like domain and HAD fold in T6PP.** A sequence alignment of the T6PP enzymes from several nematode species reveals a variable N-terminus and conserved MIT domain. A high degree of conservation is seen in the HAD domain. The structure outline from *B. malayi* is depicted as helices, loops, or β-strands.
(PDF)

Figure S4 **CD spectra of purified** ***B. malayi*** **T6PP and Δ59-T6PP.** CD spectra of purified *B. malayi* T6PP and Δ59-T6PP were collected to ensure the proteins were well-folded. Removal of the first 59 residues does not affect the folding or activity of the enzyme.
(TIF)

Figure S5 **Sequence alignments of nematode and** ***Mycobacterium*** **T6PP enzymes.** A sequence alignment of the T6PP enzymes from nematode and *Mycobacterium* reveals the conservation of the N-terminal MIT domain. The conservation of this domain was found only in T6PP enzymes from nematodes and members of the *Mycobacterium* genus and not in T6PP of bacterial origin.
(PDF)

Figure S6 **Sequence alignments of T6PP enzymes from a range of organisms.** A sequence alignment of selected T6PP enzymes from a range of organisms reveals that the MIT-like domain is found only in nematodes and the *Mycobacterium* genus.
(PDF)

Figure S7 **Analysis of the MIT-like domain from** ***B. malayi*** **T6PP.** Thermal stability of the *B. malayi* Δ59-T6PP reveals a strong interaction between the MIT and HAD domains. Thermofluor analysis of *B. malayi* Δ59-T6PP using SYPRO Orange revealed a two-state transition (A) in contrast to the single transition seen by CD (Figure 3C). Treatment with trypsin at 25°C revealed a stable enzyme, while treatment at 45°C revealed degradation products of approximately 34-, 27-, 12- and 10-kDa. (B). MS analysis of these fragments revealed instability of the C-terminus (34-kDa fragment = protein minus orange-red, 27-kDa fragment = protein minus orange-red and neon green, 12-kDa fragment = orange-red and 10-kDa fragment = neon green (C)). These results suggest that the interaction between the MIT domain and the HAD core is relatively stable. Analysis of the MIT and HAD domains (expressed as separate clones or as part of a pET-DUET vector) reveals that the MIT domain is stable one its own (D). d179+1–178 (pET-DUET), d179 or 1–178 were expressed (uninduced –un or induced –in) and tested for solubility (T –total cell fraction, S –soluble fraction). The MIT domain is stable and soluble as a standalone domain, whereas the HAD domain is not.
(TIF)

Figure S8 **Essential residues in Bm T6PP are conserved in T6PP enzymes from other organisms.** Conservatively replaced residues in other T6PP enzymes were identified using the structure from *T. acidophilum* (A) or homology models generated by the Phyre2 server using *T. acidophilum* coordinates as the template. Due to differences in length in the cap domains among the enzymes, sequence alignments were not appropriate to identify conserved residues. Homology models were generated for *Mycobacterium tuberculosis* (B), *Saccharomyces cerevisiae* (C) and *Escherichia coli* (D).
(TIF)

Figure S9 **FTMap- and mutagenesis-guided model of the T6PP/trehalose 6-phosphate complex.** The *B. malayi* cap was placed in the closed conformation by superposition with the closed form from *T. acidophilum*. DynDom analysis of the proposed closed model of T6PP reveal a 45.6° rotation of the cap domain with respect to the core. The original cap is shown in gold, and the *T. acidophilum* oriented cap position is colored cyan (A). In this model, the residues identified as important for binding and/or catalysis are labeled and are found in close proximity to the proposed trehalose 6-phosphate binding pocket (B).
(TIF)

Table S1 **Elimination of** ***tps-1*** **function suppresses the larval arrest caused by** ***gob-1*** **RNAi.**
(DOCX)

Table S2 **List of cloning and mutagenesis primers used in this study.**
(DOCX)

Table S3 **Crystallographic and refinement statistics for** ***B. malayi*** **T6PP data sets.**
(DOCX)
