# Supplementary figures and images for: Structure of the Trehalose-6-phosphate Phosphatase from Brugia malayi Reveals Key Design Principles for Anthelmintic Drugs
Source: PLoS Pathog. 2014 Jul 3;10(7):e1004245. doi: 10.1371/journal.ppat.1004245 (PMC4081830; doi:10.1371/journal.ppat.1004245)

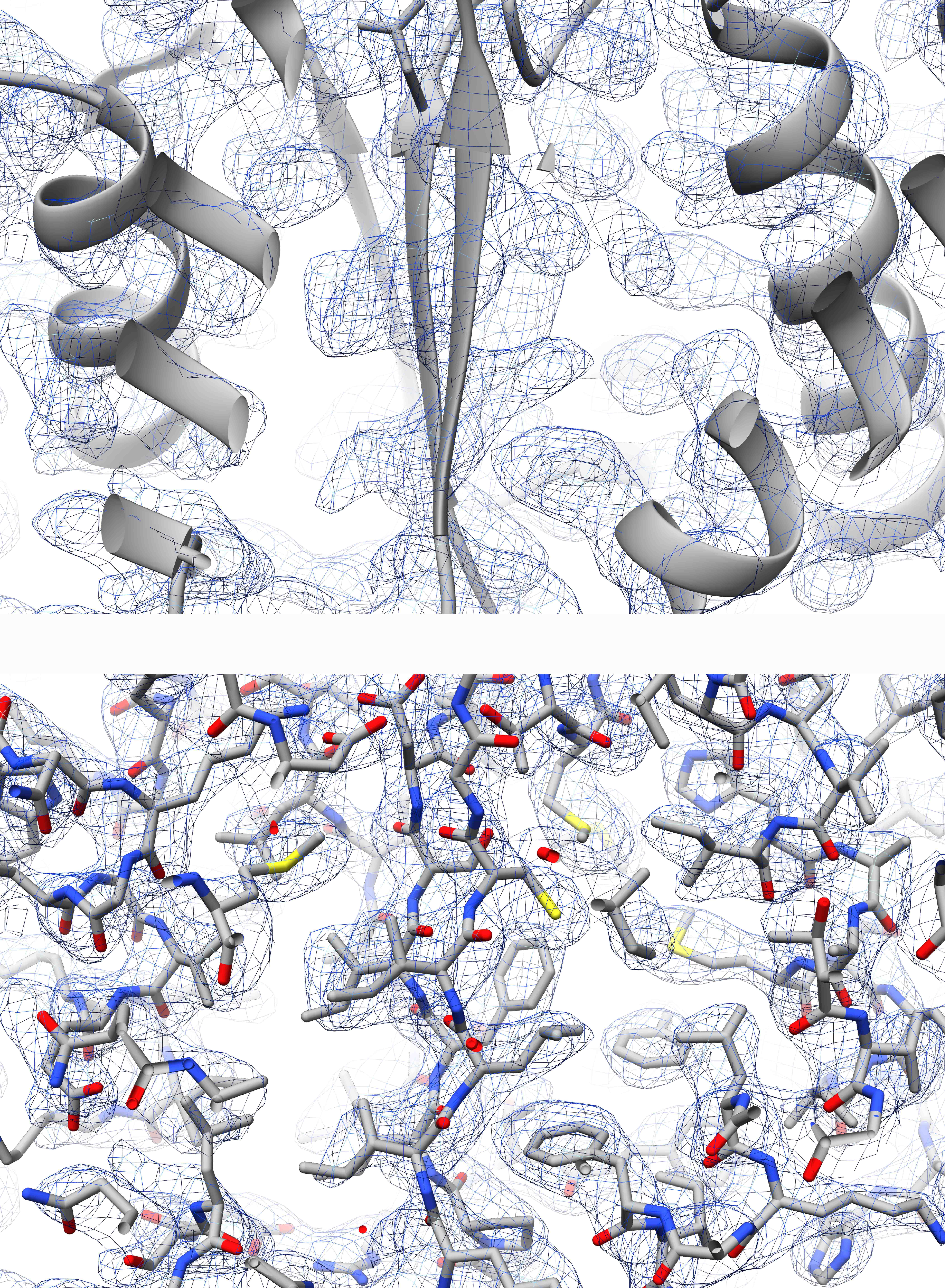

Supplement: Figure S1 — 2Fo-Fc electron density maps for Bm T6PP countered at 1.5σ. Maps were calculated by manually omitting the core β-sheet of the Rossmann fold and carrying out a round of simulated annealing. Both panels show the same region in either ribbon form (above) or sticks (below). (TIF) [file ppat.1004245.s001.tif]

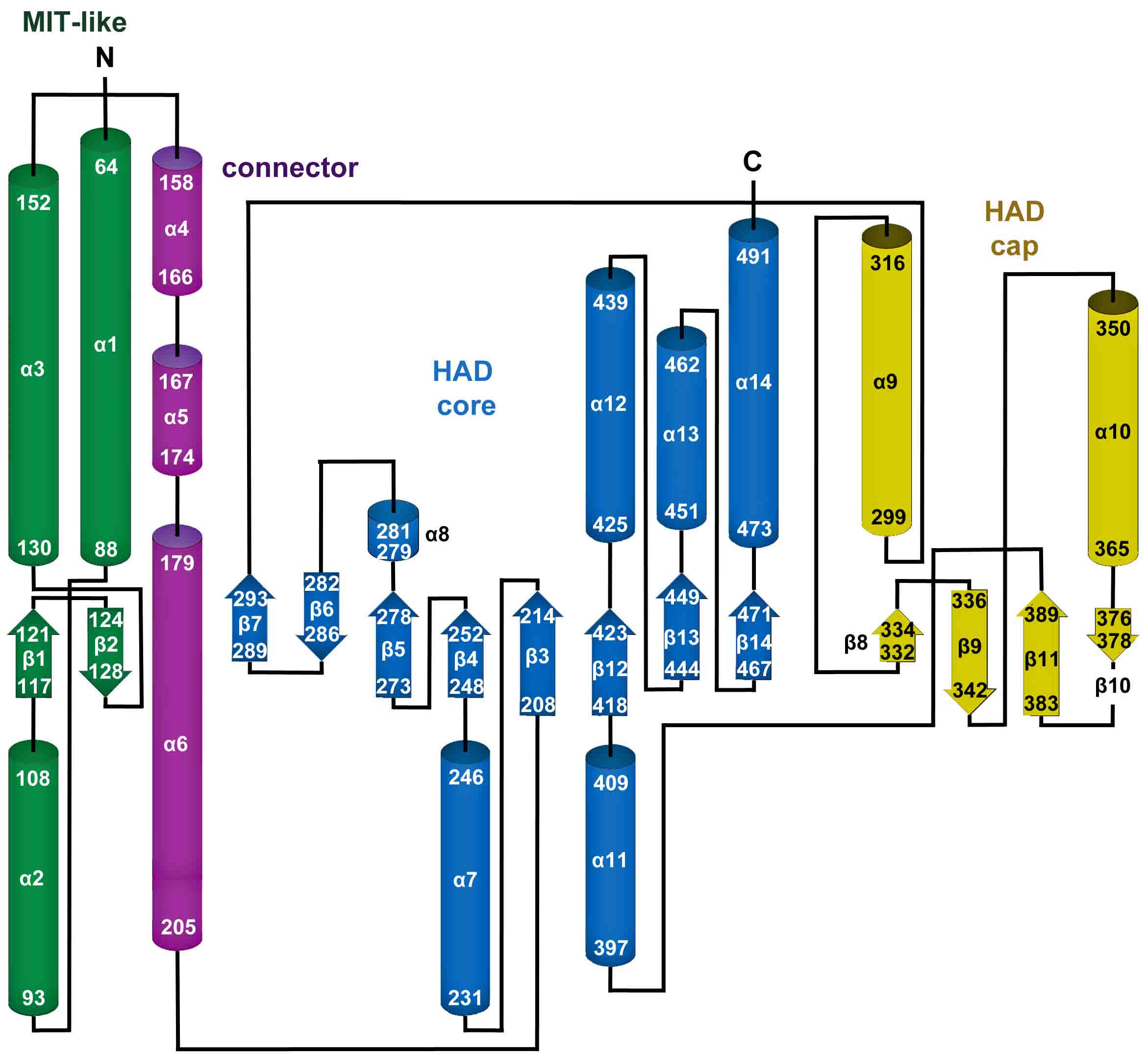

Supplement: Figure S2 — Topology model for Bm T6PP. A modified topology diagram from the Topsan server (http://www.topsan.org) is depicted with the same coloring scheme as the structure in Figure 2. (TIF) [file ppat.1004245.s002.tif]

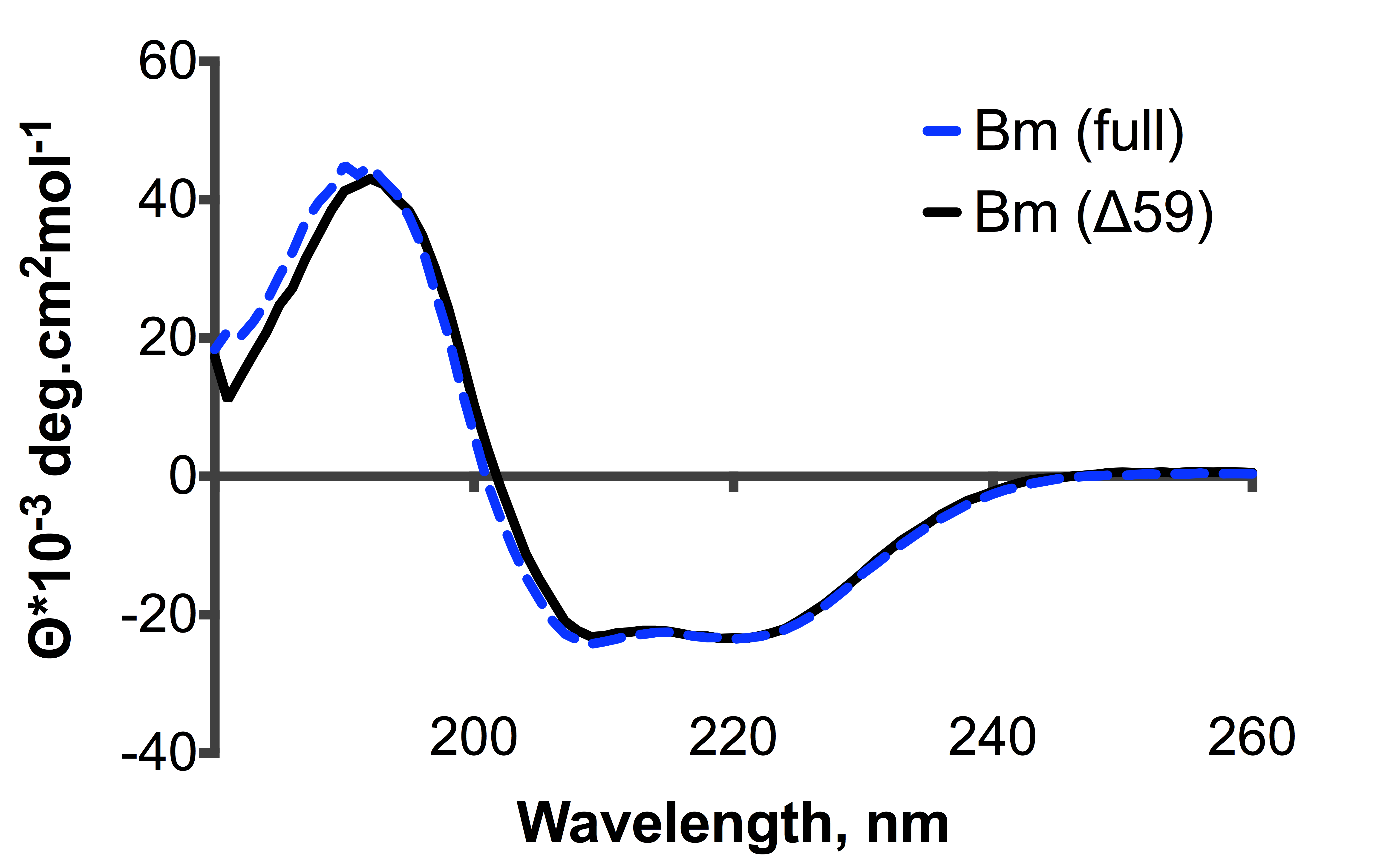

Supplement: Figure S4 — CD spectra of purified B. malayi T6PP and Δ59-T6PP. CD spectra of purified B. malayi T6PP and Δ59-T6PP were collected to ensure the proteins were well-folded. Removal of the first 59 residues does not affect the folding or activity of the enzyme. (TIF) [file ppat.1004245.s004.tif]

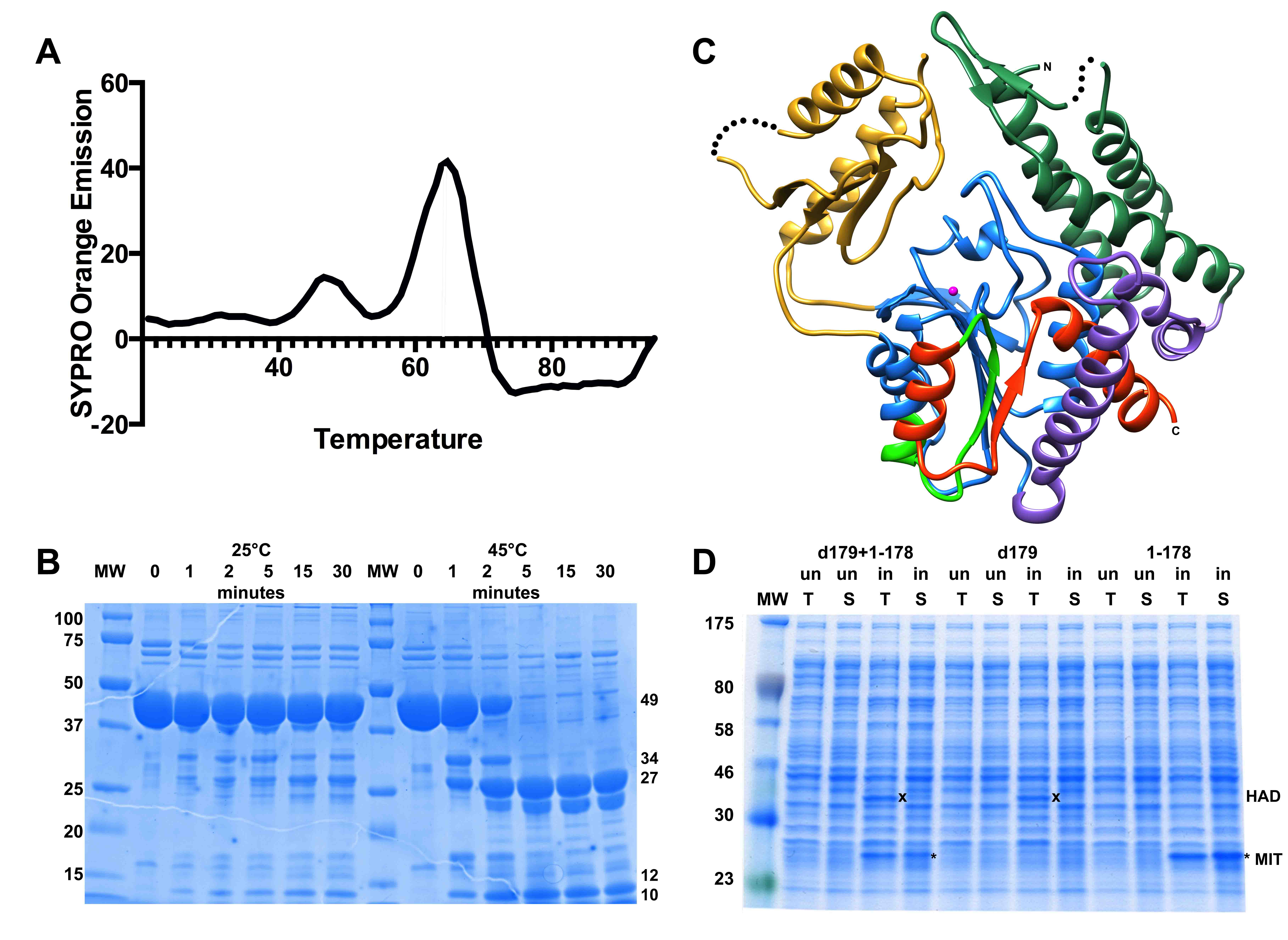

Supplement: Figure S7 — Analysis of the MIT-like domain from B. malayi T6PP. Thermal stability of the B. malayi Δ59-T6PP reveals a strong interaction between the MIT and HAD domains. Thermofluor analysis of B. malayi Δ59-T6PP using SYPRO Orange revealed a two-state transition (A) in contrast to the single transition seen by CD (Figure 3C). Treatment with trypsin at 25°C revealed a stable enzyme, while treatment at 45°C revealed degradation products of approximately 34-, 27-, 12- and 10-kDa. (B). MS analysis of these fragments revealed instability of the C-terminus (34-kDa fragment = protein minus orange-red, 27-kDa fragment = protein minus orange-red and neon green, 12-kDa fragment = orange-red and 10-kDa fragment = neon green (C)). These results suggest that the interaction between the MIT domain and the HAD core is relatively stable. Analysis of the MIT and HAD domains (expressed as separate clones or as part of a pET-DUET vector) reveals that the MIT domain is stable one its own (D). d179+1–178 (pET-DUET), d179 or 1–178 were expressed (uninduced –un or induced –in) and tested for solubility (T –total cell fraction, S –soluble fraction). The MIT domain is stable and soluble as a standalone domain, whereas the HAD domain is not. (TIF) [file ppat.1004245.s007.tif]

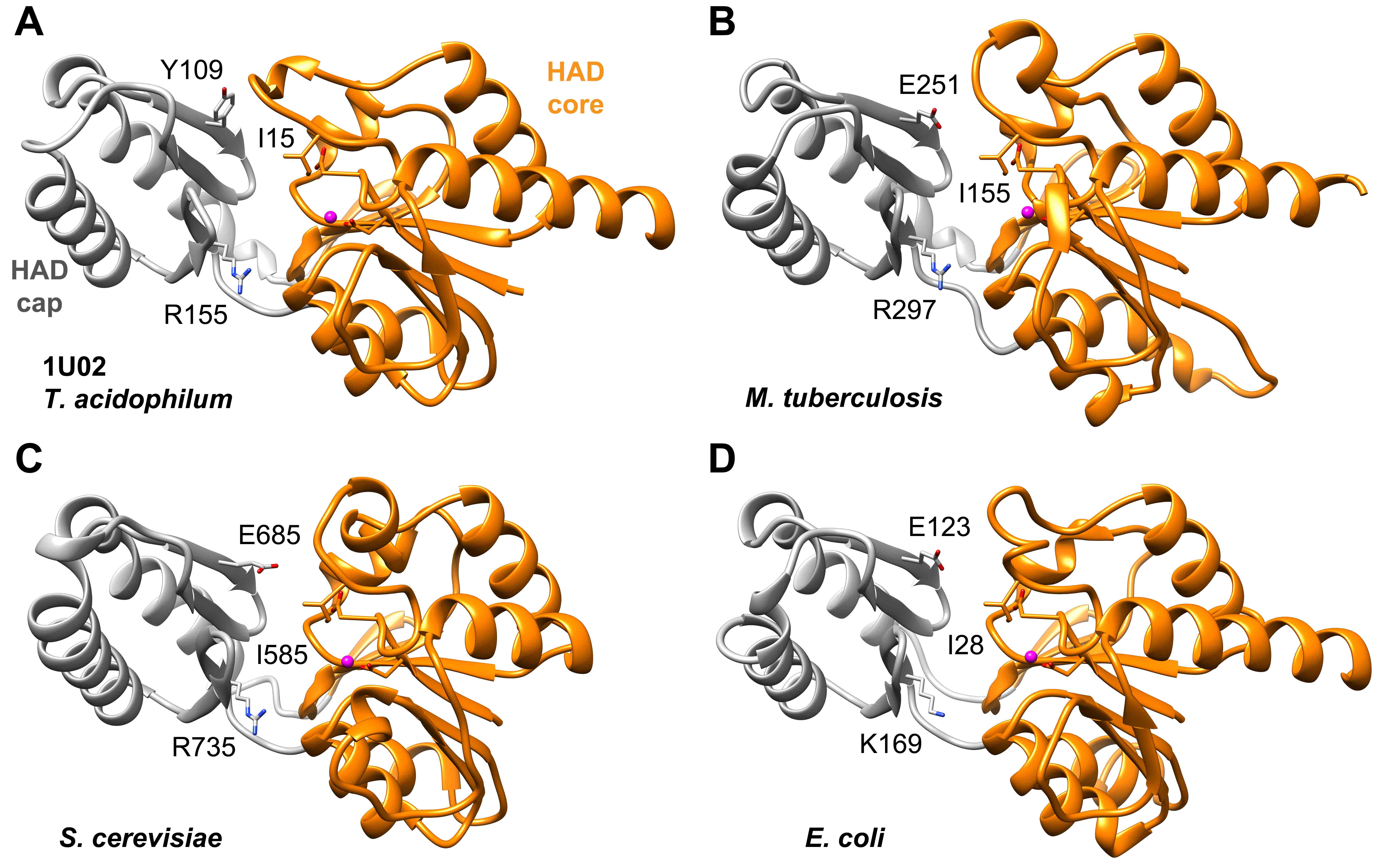

Supplement: Figure S8 — Essential residues in Bm T6PP are conserved in T6PP enzymes from other organisms. Conservatively replaced residues in other T6PP enzymes were identified using the structure from T. acidophilum (A) or homology models generated by the Phyre2 server using T. acidophilum coordinates as the template. Due to differences in length in the cap domains among the enzymes, sequence alignments were not appropriate to identify conserved residues. Homology models were generated for Mycobacterium tuberculosis (B), Saccharomyces cerevisiae (C) and Escherichia coli (D). (TIF) [file ppat.1004245.s008.tif]

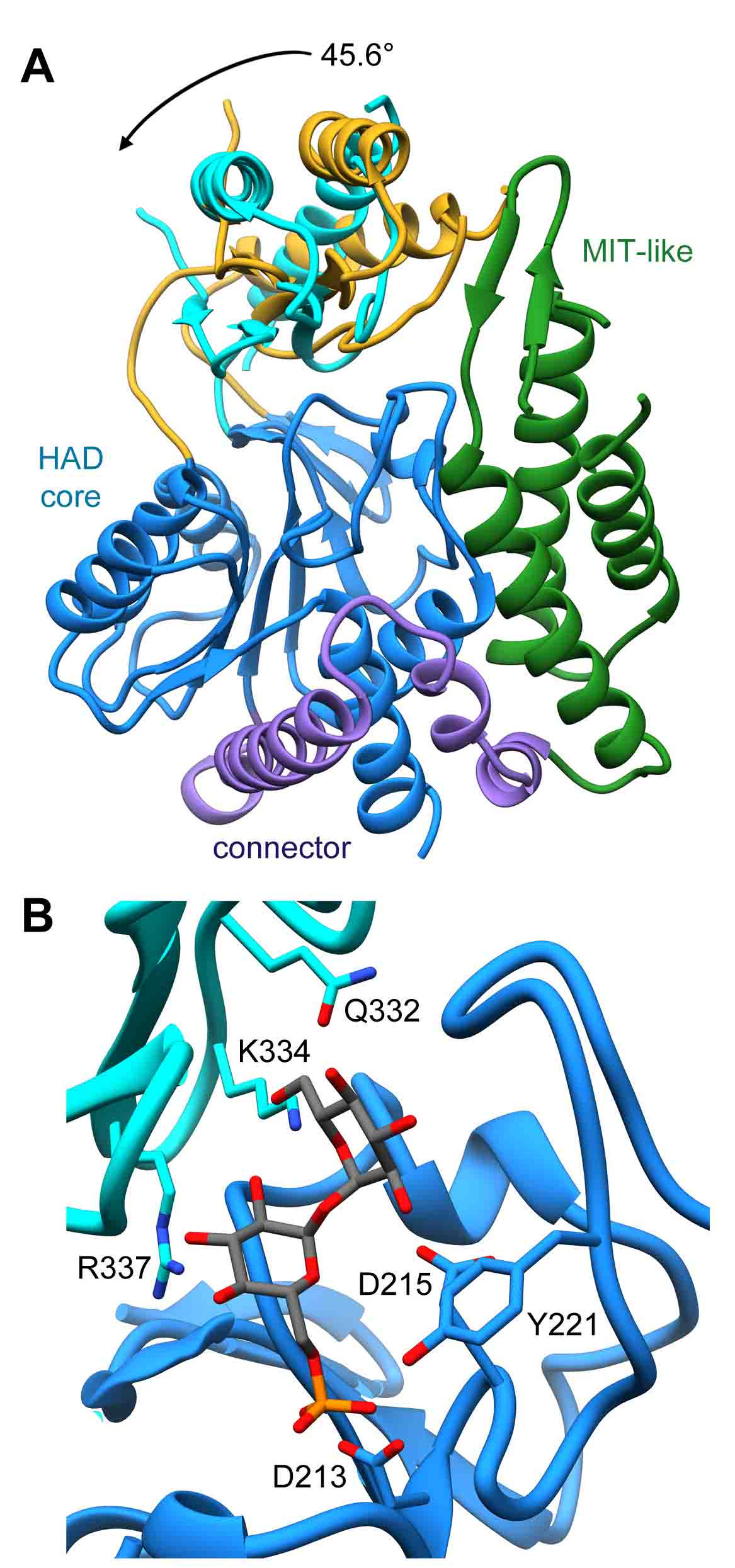

Supplement: Figure S9 — FTMap- and mutagenesis-guided model of the T6PP/trehalose 6-phosphate complex. The B. malayi cap was placed in the closed conformation by superposition with the closed form from T. acidophilum. DynDom analysis of the proposed closed model of T6PP reveal a 45.6° rotation of the cap domain with respect to the core. The original cap is shown in gold, and the T. acidophilum oriented cap position is colored cyan (A). In this model, the residues identified as important for binding and/or catalysis are labeled and are found in close proximity to the proposed trehalose 6-phosphate binding pocket (B). (TIF) [file ppat.1004245.s009.tif]
