# Supplementary material for: Structure of the Trehalose-6-phosphate Phosphatase from Brugia malayi Reveals Key Design Principles for Anthelmintic Drugs
Source: PLoS Pathog. 2014 Jul 3;10(7):e1004245. doi: 10.1371/journal.ppat.1004245 (PMC4081830; doi:10.1371/journal.ppat.1004245)
Supplement: Figure S3 — A variable N-terminus and conserved MIT-like domain and HAD fold in T6PP. A sequence alignment of the T6PP enzymes from several nematode species reveals a variable N-terminus and conserved MIT domain. A high degree of conservation is seen in the HAD domain. The structure outline from B. malayi is depicted as helices, loops, or β-strands. (PDF) [file ppat.1004245.s003.pdf]

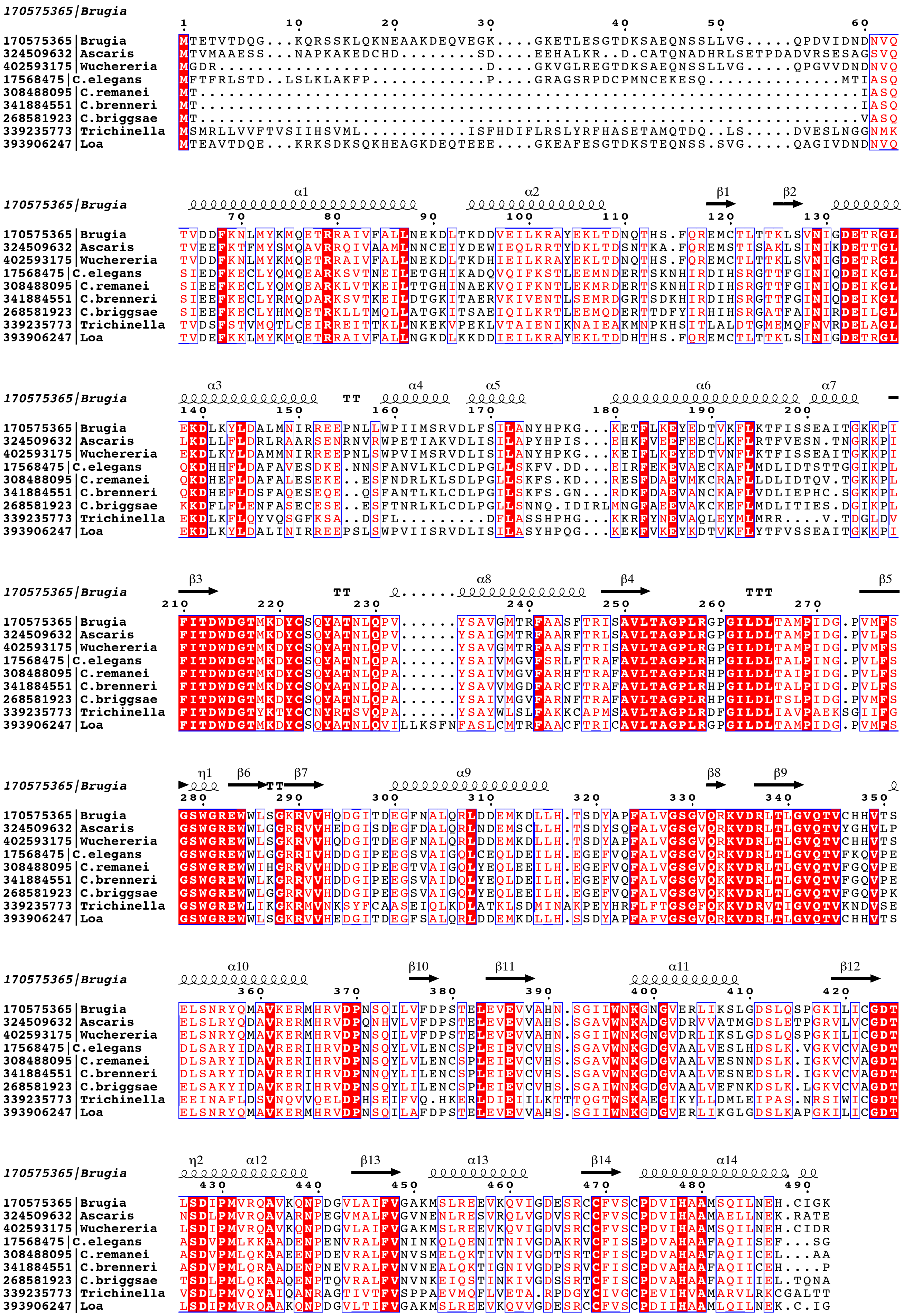
170575365|Brugia
1 10 20 30 40 50 60
170575365|Brugia MTETVTDQG...KQRSSKLQKNEAAKDEQVEGK....GKETLESGTDKSAEQNSSLLVG.....QPDVIDNDNVQ
324509632|Ascaris MTVMAAESS...NAPKAKEDCHD........SD....EEHALKR..D.CATQNADHRLSETPDADVRSESAGSVQ
402593175|Wuchereria MGDR............................D....GKVGLREGTDKSAEQNSSLLVG.....QPGVVDNDNVQ
17568475|C.elegans MFTFRLSTD..LSLKLAKFP............P....GRAGSRPDCPMNCEKESQ..............MTIASQ
308488095|C.remanei MT...IASQ
341884551|C.brenneri MT...IASQ
268581923|C.briggsae MT...VASQ
339235773|Trichinella MSMRLLVVFTVSIIHSVML...........ISFHDIFLRSLYRFHASETAMQTDQ..LS.....DVESLNGGNMK
393906247|Loa MTEAVTDQE...KRKSDKSQKHEAGKDEQTEEE....GKEAFESGTDKSTEQNSS.SVG.....QAGIVDNDNVQ
170575365|Brugia
α1 α2 β1 β2
70 80 90 100 110 120 130
170575365|Brugia TVDDFKNLMYKMQETRRAIVFALLNEKDLTKDDVEILKRAYEKLTDNQTHS.FQREMCTLTTKLSVNIGDETRGL
324509632|Ascaris TVEEFKTFMYSMQAVRRQIVAAMLNNCEIYDEWIEQLRRTYDKLTDSNTKA.FQREMSTISAKLSINIKDETTGL
402593175|Wuchereria TVDDFKNLMYKMQETRRAIVFALLNEKDLTKDHIEILKRAYEKLTDNQTHS.FQREMCTLTTKLSVNIGDETRGL
17568475|C.elegans SIEDFKECLYQMQEARKSVTNEILETGHIKADQVQIFKSTLEEMNDERTSKNHIRDIHSRGTTFGINIQDEIKGL
308488095|C.remanei SIEEFKECLYQMQEARKLVTKEILTTGHINAEKVQIFKNTLEKMRDERTSKNHIRDIHSRGTTFGINIQDEIKGL
341884551|C.brenneri SIEEFKECLYRMQDARKSVTKEILDTGKITAERVKIVENTLSEMRDGRTSDKHIRDIHSRGTTFGINIQDEIKGL
268581923|C.briggsae SIEEFKECLYHMQETRKLLTMQLLATGKITSAEIQILKRTLEEMQDERTTDFYIRHIHSRGATFAINIRDEILGL
339235773|Trichinella TVDSFSTVMQTLCEIRREITTKLLNKEKVPEKLVTAIENIKNAIEAKMNPKHSITLALDTGMEMQFNVRDELAGL
393906247|Loa TVDEFKKLMYKMQETRRAIVFALLNGKDLKKDDIEILKRAYEKLTDDHTHS.FQREMCTLTTKLSINIGDETRGL
170575365|Brugia
α3 TT α4 α5 α6 α7
140 150 160 170 180 190 200
170575365|Brugia EKDLKYLDALMNIRREEPNLLWPIIMSRVDLFSILANYHPKG...KETFLKEYEDTVKFLKTFISSEAITGKKPI
324509632|Ascaris LKDLLFLDRLRAARSENRNVRWPETIAKVDLISILAPYHPIS...EHKFVEEFEECLKFLRTFVESN.TNGRKPI
402593175|Wuchereria EKDLKYLDAMMNIRREEPNLSWPVIMSRVDLISILANYHPKG...KEIFLKEYEDTVNFLKTFISSEAITGKKPI
17568475|C.elegans QKDHHFLDAFAVESDKE.NNSFANVLKLCDLPGLLSKFV.DD...EIRFEKEVAECKAFLMDLIDTSTTGGIKPL
308488095|C.remanei QKDHEFLDAFALESEKE..ESFNDRLKLSDLPGLLSKFS.KD...RESFDAEVMKCRAFLLDLIDTQV.TGKKPL
341884551|C.brenneri QKDHEFLDSFAQESEQE..QSFANTLKLCDLPGLLSKFS.GN...RDKFDAEVANCKAFLVDLIEPHC.SGKKPL
268581923|C.briggsae KKDFLFLENFASECESE..ESFTNRLKLCDLPGLLSNNQ.IDIRLMNGFAEEVAKCKEFLMDLITIES.DGIKPL
339235773|Trichinella EKDLKFLQYVQSGFKSA..DSFL.........DFLASSHPHG...KKRFYNEVAQLEYMLMRR....V.TDGLDV
393906247|Loa EKDLKYLDALINIRREEPSLSWPVIISRVDLISILASYHPQG...KEKFVKEYKDTVKFLYTFVSSEAITGKKPI
170575365|Brugia
β3 TT α8 β4 TTT β5
210 220 230 240 250 260 270
170575365|Brugia FITDWDGTMKDYCSQYATNLQPV......YSAVGMTRFAASFTRISAVLTAGPLRGPGILDLTAMPIDG.PVMFS
324509632|Ascaris FITDWDGTMKDYCSQYATNLQPV......YSAIGMTRFAARFTRLSAVLTAGPLRGPGILDLTAMPIDG.PVLFS
402593175|Wuchereria FITDWDGTMKDYCSQYATNLQPV......YSAVGMTRFAASFTRISAVLTAGPLRGPGILDLTAMPIDG.PVMFS
17568475|C.elegans FITDWDGTMKDYCSQYATNLQPA......YSAIVMGVFSRLFTRAFAVLTAGPLRHPGILDLTALPING.PVLFS
308488095|C.remanei FITDWDGTMKDYCSQYATNLQPA......YSAIVMGVFARHFTRAFAVLTAGPLRHPGILDLTAIPIDG.PVLFS
341884551|C.brenneri FITDWDGTMKDYCSQYATNLQPA......YSAVVMGDFARCFTRAFAVLTAGPLRHPGILDLTALPIDG.PVLFS
268581923|C.briggsae FITDWDGTMKDYCSQYATNLQPA......YSAIVMGVFARNFTRAFAVLTAGPLRHPGILDLTSLPIDG.PVMFS
339235773|Trichinella FITDWDGTYKTYCNYRTSVQPA......YSAYWLSLFAKKCAPMSAVLTAGPLRDFGILDLIAVPAEKSGIIFG
393906247|Loa FITDWDGTMKDYCSQYATNLQVILLKSFNFASLCMTRFAACFTRICAVLTAGPLRGPGILDLTAMPIDG.PVMFS
170575365|Brugia
η1 β6 TT β7 α9 β8 β9
280 290 300 310 320 330 340 350
170575365|Brugia GSWGREWWLSGKRVVHQDGITDEGFNALQRLDDEMKDLLH.TSDYAPFALVGSGVQRKVDRLTLGVQTVCHHVTS
324509632|Ascaris GSWGREWWLGGRRVVHEDGISDEGFDALQRLNDEMSCLLH.TSDYSQFALVGSGVQRKVDRLTLGVQTVYGHVLP
402593175|Wuchereria GSWGREWWLSGKRVVHEDGITDEGFNALQRLDDEMKDLLH.TSDYAPFALVGSGVQRKVDRLTLGVQTVCHHVTS
17568475|C.elegans GSWGREWWLGGRRIVHDDGIPEEGSVAIGQLCEQLDEILH.EGEFVQFALVGSGVQRKVDRLTLGVQTVFKQVPE
308488095|C.remanei GSWGREWWIHGRRVVHDDGIPEEGTVAIGQLYEQLEEILH.EGEFVQFALVGSGVQKKVDRLTLGVQTVFGQVPE
341884551|C.brenneri GSWGREWWLKGRRVVHDDGIPEEGSVAIDQLYEQLDEILH.EGEFVQFALVGSGVQKKVDRLTLGVQTVFGQVPE
268581923|C.briggsae GSWGREWWLGGRRVVHDDGIPEEGSVAIGQLYEQLEEILH.EGEFVQFALVGSGVQKKVDRLTLGVQTVFGQVPK
339235773|Trichinella GSWGREWLIKGKRMVNKSYFCAASEIQLKDLATKLSDMINAKPEYHRFLFTGSGFQKKVDRVTIGVQTVKNDVSE
393906247|Loa GSWGREWWLSGKRVVHEDGITDEGFSALQRLDDEMKDLLH.SSDYAPFAFVGSGVQRKVDRLTLGVQTVCHHVTS
170575365|Brugia
α10 β10 β11 α11 β12
360 370 380 390 400 410 420
170575365|Brugia ELSNRYQMAVKERMHRVDPNSQILVFDPSTELEVEVVAHN.SGIIWNKGNGVERLIKSLGDSLQSPGKILICGDT
324509632|Ascaris ELSLRYQDAVRERMHRVDPQNHVLVFDPSTELEVEVVAHS.SGAVWNKADGVDRVVATMGDSLETPGRVLVCGDT
402593175|Wuchereria ELSNRYQMAVKERMHRVDPNSQILVFDPSTELEVEVVAHN.SGIIWNKGNGVDRLIKSLGDSLQSPGKILICGDT
17568475|C.elegans DLSARYIDAVRERIHRVDPNSQYLVLENCSPLEIEVCVHS.SGAVWNKGDGVAALVESLHDSLK.VGKVCVAGDT
308488095|C.remanei DLSARYIDAVRERIHRVDPNNQYLILENCSPLEIEVCVHS.SGAVWNKGDGVAALVESNNDSLK.IGKVCVAGDT
341884551|C.brenneri DLSARYIDAVRERIHRVDPNSQYLVLENCSPLEIEVCVHS.SGAVWNKGDGVAALVESNEDSLR.IGKVCVAGDT
268581923|C.briggsae ELSAKYIDAVKERIHRVDPNSQYLILENCSPLEIEVCVHS.SGAIWNKGDGVAALVEFNKDSLK.LGKVCVAGDT
339235773|Trichinella EEINAFLDSVNQVVQELDPHSEIFVQ.HKERLDIEIILKTTQGTWSKAEGIKYLLDMLEIPAS.NRSIWICGDT
393906247|Loa ELSNRYQMAVKERMHRVDPNSQILAFDPSTELEVEVVAHS.SGIIWNKGDGVERLIKGLGDSLKAPGKILICGDT
170575365|Brugia
η2 α12 β13 α13 β14 α14
430 440 450 460 470 480 490
170575365|Brugia LSDIPMVRQAVKQNPDGVLAIFVGAKMSLREEVKQVIGDESRCCFVSCPDVIHAAMSQILNEH.CIGK
324509632|Ascaris NSDLPMVRQAVARNPEGVMALFVGVNENLRESVRQLVGDVSRCCFVSCPDVIHAAMAELLNEK.RATE
402593175|Wuchereria LSDIPMVRQAVKQNPDGVLAIFVGAKMSLREEVKQVIGDVSRCCFVSCPDVIHAAMSQILNEH.CIDR
17568475|C.elegans ASDVPMLKKAADENPENVRALFVNINKKQLQENITNIVGDAKRVCFISSPDVAHAAFAQIISEF...SG
308488095|C.remanei ASDVPMLQKAAEENPDEVRALFVNVSMELQKTIVNIVGDTSRTCFISCPDVAHAAFAQIICEL...AA
341884551|C.brenneri ASDVPMLQRAADENPNEVRALFVNVNEALQKTIGNIVGDPSRVCFISCPDVAHAAFAQIICEH....P
268581923|C.briggsae TSDLPMLQKAAQENPTQVRALFVNVNKEIQSTINKIVGDSSRTCFISCPDVAHAAFAQIIIEL.TQNA
339235773|Trichinella VSDLPMVQYAIQANRAGTIVTFVSPPAEVMQFLVETA.RPDGYCIVGCPEVIHVAMARVLRKCGALTT
393906247|Loa LSDIPMVRQAAKQNPDGVLTIFVGAKMSLREEVKQVVGDESRCCFVSCPDIIHAAMLQILNEK.C.IG
