# Supplementary material for: Structure of the Trehalose-6-phosphate Phosphatase from Brugia malayi Reveals Key Design Principles for Anthelmintic Drugs
Source: PLoS Pathog. 2014 Jul 3;10(7):e1004245. doi: 10.1371/journal.ppat.1004245 (PMC4081830; doi:10.1371/journal.ppat.1004245)
Supplement: Figure S5 — Sequence alignments of nematode and Mycobacterium T6PP enzymes. A sequence alignment of the T6PP enzymes from nematode and Mycobacterium reveals the conservation of the N-terminal MIT domain. The conservation of this domain was found only in T6PP enzymes from nematodes and members of the Mycobacterium genus and not in T6PP of bacterial origin. (PDF) [file ppat.1004245.s005.pdf]

```
170575365|Brugia
                              1        10        20        30        40        50
170575365|Brugia            MTETVTDQ...GKQRSS.KLQK.NEAA.KDEQVEGKGKETLESGTDKSAEQN..S..SLLVGQPD.VIDN
324509632|Ascaris           MTVMAAESSNAPKAKEDCHDSD.EEHA..............LKRDCAT.QNADHRLSETPDADVRSESA
402593175|Wuchereria        MGDRDG..........................KVGLREGTDKSAEQN..S..SLLVGQPG.VVDN
17568475|C.elegans          MFTFRLST.....DLSL.KL..........AKFPPGRAGSRPDCPMNCEKE.............SQMT
308488095|C.remanei         MTI...................................................................
341884551|C.brenneri        MTI...................................................................
268581923|C.briggsae        MTV...................................................................
339235773|Trichinella       MSMRLLVVFTVSIIHSV.MLISFHDIFLRSLYRFHASET.........AMQT..D..QL..SDVE.SLNG
393906247|Loa               MTEAVTDQ...EKRKSD.KSQK.HEAG.KDEQTEEEGKEAFESGTDKSTEQN.....SSSVGQAG.IVDN
15610508|M.tuberculosis     MRKLGPVT.IDPRRHDA.VLFD................................................
340628353|M.canetti         MRKLGPVT.IDPRRHDA.VLFD................................................
15827129|M.leprae           MPV....T.IDPRRHSA.ALFD.LDA...........................................V
183981178|M.marinum         MSV....T.IDPRRHDA.VLFD................................................
443489644|M.liflandii       MSV....T.IDPRRHDA.VLFD................................................
118616688|M.ulcerans        MSV....T.IDPRRHDA.VLFD................................................
495048542|M.colombiense     MGKSGPAV.IDPRRHDA.VLFD................................................
379756214|M.intracellulare  MPV....I.IDPRRHDA.VLFD................................................
41409576|M.avium            MGESGPVV.IDPRRHDA.VLFG................................................

                                   α1                                           α2
170575365|Brugia
                            60        70        80        90                        100
170575365|Brugia            DNVQTVDDFKNLMYKMQETRRAIVFALLNEKDLT...................KDDVEILKRAYEKLTD
324509632|Ascaris           GSVQTVEEFKTFMYSMQAVRRQIVAAMLNNCEIY...................DEWIEQLRRTYDKLTD
402593175|Wuchereria        DNVQTVDDFKNLMYKMQETRRAIVFALLNEKDLT...................KDHIEILKRAYEKLTD
17568475|C.elegans          IASQSIEDFKECLYQMQEARKSVTNEILETGHIK...................ADQVQIFKSTLEEMND
308488095|C.remanei         .ASQSIEEFKECLYQMQEARKLVTKEILTTGHIN...................AEKVQIFKNTLEKMRD
341884551|C.brenneri        .ASQSIEEFKECLYRMQDARKSVTKEILDTGKIT...................AERVKIVENTLSEMRD
268581923|C.briggsae        .ASQSIEEFKECLYHMQETRKLLTMQLLATGKIT...................SAEIQILKRTLEEMQD
339235773|Trichinella       GNMKTVDSFSTVMQTLCEIRREITTKLLNKEKVP...................EKLVTAIENIKNAIEA
393906247|Loa               DNVQTVDEFKKLMYKMQETRRAIVFALLNGKDLK...................KDDIEILKRAYEKLTD
15610508|M.tuberculosis     ...TTLDATQELVRQLQEVGVG.TGVFGSGL.........................DVPIVAAGRLAV
340628353|M.canetti         ...TTLDATQELVRQLQEVGVG.TGVFGSGL.........................DVPIVAAGRLAV
15827129|M.leprae           VTDTPLDSTVTLVRQLQGIGVG.TAVFSTNRNSQGVLTATGLDHLFPVHVDGLASGKVTILVAAANRLMA
183981178|M.marinum         ...TALNSTQALVRQLQQARVG.TATFASGGGGH...................DAAIQALTESADRVGA
443489644|M.liflandii       ...TALNSTQALVRQLQQARVG.TATFASGGGGH...................DAAIQALTESADRVGA
118616688|M.ulcerans        ...TALNSTQALVRQLQQARVG.TATFASGGGGH...................DAAIQALIESADRVGA
495048542|M.colombiense     ...DEAALGSTLAVRLRETGLG.TAVIAAHD..........................PAQAADRLQV
379756214|M.intracellulare  ...LETDLGSTLAARLRDVGVG.TAVVPADG..........................PARAAADLKV
41409576|M.avium            ...VGDALGSALASQLGQIGVG.TAAFAADG..........................PAAAADRLRV

                                      β1            β2          α3                       TT    α4
170575365|Brugia
                            110       120                 130       140       150       160
170575365|Brugia            NQTHS.FQREMC......TLTTK....LSVNIGDETRGL.EKDLK..YLDALMNIRREEPNLLWPIIMSR
324509632|Ascaris           SNTKA.FQREMS......TISAK....LSINIKDETTGL.LKDLL..FLDRLRAARSENRNVRWPETIAK
402593175|Wuchereria        NQTHS.FQREMC......TLTTK....LSVNIGDETRGL.EKDLK..YLDAMMNIRREEPNLSWPVIMSR
17568475|C.elegans          ERTSKNHIRDIH......SRGTT....FGINIQDEIKGL.QKDHH..FLDAFAVESDKE.NNSFANVLKL
308488095|C.remanei         ERTSKNHIRDIH......SRGTT....FGINIQDEIKGL.QKDHE..FLDAFALESEKE..ESFNDRLKL
341884551|C.brenneri        GRTSDKHIRDIH......SRGTT....FGINIQDEIKGL.QKDHE..FLDSFAQESEQE..QSFANTLKL
268581923|C.briggsae        ERTTDFYIRHIH......SRGAT....FAINIRDEILGL.KKDFL..FLENFASECESE..ESFTNRLKL
339235773|Trichinella       KMNPKHSITLAL......DTGME....MQFNVRDELAGL.EKDLK..FLQYVQSGFKSA..DSF......
393906247|Loa               DHTHS.FQREMC......TLTTK....LSINIGDETRGL.EKDLK..YLDALINIRREEPSLSWPVIISR
15610508|M.tuberculosis     .RPGRCVVVSAHSAGVTAARESGFALI....IGVDRTGC.RDALRRDGADTVVTD...............
340628353|M.canetti         .RPGRCVVVSAHSAGVTAARESGFALI....IGVDRTGC.RDALRRDGADTVVTD...............
15827129|M.leprae           .QPGRCVVVAVDAAGITAARYGGFALVLGLVIGEDQTGH.RDTLRNSGADTVVAD...............
183981178|M.marinum         .RPGRCVVITADAASVAAARDSGFALV....IGVDQAGH.RDALPDHGADTVLAD...............
443489644|M.liflandii       .RPGRCVVITADAASVAAARDSGFALV....IGVDQAGH.RDALPDHGADTVLAD...............
118616688|M.ulcerans        .RPGRCVVITADAASVAAARDSGFALV....IGVDQAGH.RDALPDHGADTVLAD...............
495048542|M.colombiense     .RPGRCVVVAGDAAGIEAARAAGFALV....IAVDPAGH.GETLCEAGADVVVDA...............
379756214|M.intracellulare  .RPDRAVVVAGAAAGVEAARAAGFASV....IGVGPAKS.AGALRDAGADAVVSD...............
41409576|M.avium            .RPGRCVVVAGDPAAVEAARTAGFALV....IGLAPVGRDGDGLRGAGADAVVAE...............

                              α5               α6                α7        β3                 TT
170575365|Brugia
                             170       180       190       200       210       220       230
170575365|Brugia            VDLFSILANYHP..KGKETFLKEYEDTVKFLKTFISSEAITGKKPIFITDWDGTMKDYCSQYATNLQPV.
324509632|Ascaris           VDLISILAPYHP..ISEHKFVEEFEECLKFLRTFVES.NTNGRKPIFITDWDGTMKDYCSQYATNLQPV.
402593175|Wuchereria        VDLISILANYHP..KGKEIFLKEYEDTVNFLKTFISSEAITGKKPIFITDWDGTMKDYCSQYATNLQPV.
17568475|C.elegans          CDLPGLLSKFVD...DEIRFEKEVAECKAFLMDLIDTSTTGGIKPLFITDWDGTMKDYCSQYATNLQPA.
308488095|C.remanei         SDLPGLLSKFSK...DRESFDAEVMKCRAFLLDLIDT.QVTGKKPLFITDWDGTMKDYCSQYATNLQPA.
341884551|C.brenneri        CDLPGLLSKFSG...NRDKFDAEVANCKAFLVDLIEP.HCSGKKPLFITDWDGTMKDYCSQYATNLQPA.
268581923|C.briggsae        CDLPGLLSNNQIDIRLMNGFAEEVAKCKEFLMDLITI.ESDGIKPLFITDWDGTMKDYCSQYATNLQPA.
339235773|Trichinella       ...LDFLASSHP..HGKKRFYNEVAQLEYMLMR.....RVTDGLDVFITDWDGTYKTYCCNYRTSVQPA.
393906247|Loa               VDLISILASYHP..QGKEKFVKEYKDTVKFLYTFVSSEAITGKKPIFITDWDGTMKDYCSQYATNLQVIL
15610508|M.tuberculosis     ......LSEVSV..RTGDRRMSQLPDALQAL...GLADGLVARQPAVFFDFDGTLSDIVEDPDAA.WLA.
340628353|M.canetti         ......LSEVSV..RTGDRRMSQLPDALQAL...GLADGLVARQPAVFFDFDGTLSDIVEDPDAA.WLA.
15827129|M.leprae           ......LGEVIV..RTGDRRMSELPDALQTL...GLTDDLTARQPAVFFDFDGTLSDIVDDPDSA.RPV.
183981178|M.marinum         ......LDEVRV..RAGDRHMSELPDALQAL...GRPDGLTVPRPAVFFDFDGTLSEIVDDPDAA.TPT.
443489644|M.liflandii       ......LDEVRV..RAGDRHMSELPDALQAL...GRPDGLTVPRPAVFFDFDGTLSEIVDDPDAA.TPT.
118616688|M.ulcerans        ......LDEVRV..RAGDRHMSELPDALQAL...GRPDGLTVPRPAVFFDFDGTLSEIVDDPDAA.TPT.
495048542|M.colombiense     ......LRDVTV..RTGDLRMSQLPDALQAL....ADGLADRQPAVFFDFDGTLSDIVDDPDTA.RPV.
379756214|M.intracellulare  ......LGEVGV..RTGDLRMSQLPDAMRALT..DAADGLAGRQPAVFFDFDGTLSDIVDDPDAA.RPV.
41409576|M.avium            ......LEQITV..RTGDRRMSQLPDASQALT..GGADGLAGRHPAVFFDFDGTLSDIVDDPDAA.RPV.
```

```
170575365|Brugia              α8  β4  TTT  β5  η1  β6  TT  β7
                                  240       250       260       270       280       290
170575365|Brugia              .....YSAVGMTRFAASFTRISAVLTAGPLRGPGILDLTAMPI.DGPVMFSGSWGREWWLSGKRVVHQDG
324509632|Ascaris             .....YSAIGMTRFAARFTRLSAVLTAGPLRGPGILDLTAMPI.DGPVLFSGSWGREWWLGGRRVVHEDG
402593175|Wuchereria          .....YSAVGMTRFAASFTRISAVLTAGPLRGPGILDLTAMPI.DGPVMFSGSWGREWWLSGKRVVHQDG
17568475|C.elegans            .....YSAIVMGVFSRLFTRAFAVLTAGPLRHPGILDLTALPI.NGPVLFSGSWGREWWLGGRRIVHDDG
308488095|C.remanei           .....YSAIVMGVFARHFTRAFAVLTAGPLRHPGILDLTAIPI.DGPVLFSGSWGREWWIHGRRVVHDDG
341884551|C.brenneri          .....YSAVVMGDFARCFTRAFAVLTAGPLRHPGILDLTALPI.DGPVLFSGSWGREWWLKGRRVVHDDG
268581923|C.briggsae          .....YSAIVMGVFARNFTRAFAVLTAGPLRHPGILDLTSLPI.DGPVMFSGSWGREWWLGGRRVVHDDG
339235773|Trichinella         .....YSAYWLSLFAKKCAPMSAVLTAGPLRDFGILDLIAVPAEKSGIIFGGSWGREWLIKGKRMVNKSY
393906247|Loa                 LKSFNFASLCMTRFAACFTRICAVLTAGPLRGPGILDLTAMPI.DGPVMFSGSWGREWWLSGKRVVHEDG
15610508|M.tuberculosis       .....PGAL......EALQKLAARCPIAVLSGRDLADVTQRVG.LPGIWYAGSHGFELTAP.DGTHHQND
340628353|M.canetti           .....PGAL......EALQELAARCPIAVLSGRDLADVTQRVG.LPGIWYAGSHGFELTAP.DGTHHQND
15827129|M.leprae             .....PGAT......EALQKLATHCPVAILSGRDLADVIKRIG.VPGIWYSGSHGFESTAP.DGTHHQND
183981178|M.marinum           .....AGAV......AALQQLAAQCPVAILSGRDLADVSQRVG.LPGIWYAGSHGFELTAP.DGTHHQNE
443489644|M.liflandii         .....AGAV......AALQQLAAQCPVAILSGRDLADVSQRVG.LPGIWYAGSHGFELTAP.DGTHHQNE
118616688|M.ulcerans          .....AGAV......AALQQLAAQCPVAILSGRDLADVSQRVG.LPGIWYAGSHGFELTAP.DGTHHQNE
495048542|M.colombiense       .....DGAT......EALDRLAARCPVAVLSGRDLADVTKRVG.VKGIWYAGSHGFELTAP.DGTHHQND
379756214|M.intracellulare    .....AGAT......EALAKLAAGCPVAVLSGRDLADVTNRVG.VPGIWYAGSHGFELTAP.DGTHHQND
41409576|M.avium              .....AGAT......AALTRLAARCPVAVLSGRDLADVTKRVG.VLGIWYAGSHGFELTAP.DGSHHQND
```

```
170575365|Brugia              α9  β8  β9  α10
                              300       310       320       330       340       350       360
170575365|Brugia              ITDEGFNALQRLDDEMKDLLHT.SDYAPFALVGSGVQRKVDRLTLGVQTVCHHVTSELSNRYQMAVKERM
324509632|Ascaris             ISDEGFDALQRLNDEMSCLLHT.SDYSQFALVGSGVQRKVDRLTLGVQTVYGHVLPELSLRYQDAVRERM
402593175|Wuchereria          ITDEGFNALQRLDDEMKDLLHT.SDYAPFALVGSGVQRKVDRLTLGVQTVCHHVTSELSNRYQMAVKERM
17568475|C.elegans            IPEEGSVAIGQLCEQLDEILHE.GEFVQFALVGSGVQRKVDRLTLGVQTVFKQVPEDLSARYIDAVRERI
308488095|C.remanei           IPEEGTVAIGQLYEQLEEILHE.GEFVQFALVGSGVQKKVDRLTLGVQTVFGQVPEDLSARYIDAVRERI
341884551|C.brenneri          IPEEGSVAIDQLYEQLDEILHE.GEFVQFALVGSGVQKKVDRLTLGVQTVFGQVPEDLSARYIDAVRERI
268581923|C.briggsae          IPEEGSVAIGQLYEQLEEILHE.GEFVQFALVGSGVQKKVDRLTLGVQTVFGQVPKELSAKYIDAVKERI
339235773|Trichinella         FCAASEIQLKDLATKLSDMINAKPEYHRFLFTGSGFQKKVDRVTIGVQTVKNDVSEEINAFLDSVNQVV
393906247|Loa                 ITDEGFSALQRLDDEMKDLLHS.SDYAPFAFVGSGVQRKVDRLTLGVQTVCHHVTSELSNRYQMAVKERM
15610508|M.tuberculosis       AAAAAIPVLKQAAAELRQQLGP.F......................PGVVVEHKRFGVAVHYRNAARDRV
340628353|M.canetti           AAAAAIPVLKQAAAELRQQLGP.F......................PGVVVEHKRFGVAVHYRNAARDRV
15827129|M.leprae             AAEATIPILEQAATQLRDQLGP.I......................PGVMVEHKRFGVAVHYRNVARDRV
183981178|M.marinum           AAAAAIPVLEQAAAQLRDRLGS.I......................PGVMVEHKRFGVATHYRNAARDRV
443489644|M.liflandii         AAAAAIPVLEQAAAQLRDRLGS.I......................PGVMVEHKRFGVATHYRNAARDRV
118616688|M.ulcerans          AAAAAIPVLEQAAAQLRDRLGS.I......................PGVMVEHKRFGVATHYRNAARNRV
495048542|M.colombiense       DAAAAIPVLEQAAGQLRDRLAG.I......................PGVVVEHKRFGVAVHYRNAERDRV
379756214|M.intracellulare    AAAVAIPVLEQAAARLRDQLGG.I......................PGVVVEHKRFGVAVHYRNAGRDRV
41409576|M.avium              DAAAAIPVLAQAAGRLRDELGA.I......................PGVVVEHKRFGVAVHYRNAARDRV
```

```
170575365|Brugia              β10  β11  α11  β12
                                 370       380       390       400       410       420
170575365|Brugia              HRVDPNSQI....LVFDPSTELEVEVVAH.NSGIIWNKGNGVERLIKSLGDSL....QSPGKILICGDTL
324509632|Ascaris             HRVDPQNHV....LVFDPSTELEVEVVAH.SSGAVWNKADGVDRVVATMGDSL....ETPGRVLVCGDTN
402593175|Wuchereria          HRVDPNSQI....LVFDPSTELEVEVVAH.NSGIIWNKGNGVDRLIKSLGDSL....QSPGKILICGDTL
17568475|C.elegans            HRVDPNSQY....LVLENCSPLEIEVCVH.SSGAVWNKGDGVAALVESLHDSL....KV.GKVCVAGDTA
308488095|C.remanei           HRVDPNSQY....LVLENCSPLEIEVCVH.SSGAVWNKGDGVAALVESNNDSL....KI.GKVCVAGDTA
341884551|C.brenneri          HRVDPNNQY....LILENCSPLEIEVCVH.SSGAVWNKGDGVAALVESNEDSL....RI.GKVCVAGDTA
268581923|C.briggsae          HRVDPNSQY....LILENCSPLEIEVCVH.SSGAIWNKGDGVAALVEFNKDSL....KL.GKVCVAGDTT
339235773|Trichinella         QELDPHSEI....FV.QHKERLDIEIILKTTTQGTWSKAEGIKYLLDMLEIPA....SN.RSIWICGDTV
393906247|Loa                 HRVDPNSQI....LAFDPSTELEVEVVAH.SSGIIWNKGDGVERLIKGLGDSL....KAPGKILICGDTL
15610508|M.tuberculosis       GEVAAAVRTAEQRHALRVTT..GREVIEL.RPDVDWDKGKTLLWVLDHLPHSG....SAPLVPIYLGDDI
340628353|M.canetti           GEVAAAVRTAGQRHALRVTT..GREVIEL.RPDVDWDKGKTLLWVLDHLPHSG....SAPLVPIYLGDDI
15827129|M.leprae             NEVAVAVRTAGQRNALRVTT..GREVIEL.RPDIDWDKGKTLHWVIDRLHHAGTQVGSASLMPICLGDDI
183981178|M.marinum           GEIAAVVRAAGQRDGLRVTT..GREVIEL.RPDIDWDKGKTLRWVIDHLPDQR....AAPLVPIYLGDDI
443489644|M.liflandii         GEIAAVVRAAGQRDGLRVTT..GREVIEL.HPDIDWDKGKTLRWVIDHLPDQR....AAPLVPIYLGDDI
118616688|M.ulcerans          GKIAAVVRAAGQRDGLRVTT..GREVIEL.HPDIDWDKGKTLRWVIDHLPDQR....AAPLVPIYLGDDI
495048542|M.colombiense       GEVLAAVRAAGRRDELRVTT..GREVIEL.RPDLDWDKGKTLRWVIDHLHEAG....SGPLTPVYLGDDI
379756214|M.intracellulare    GEVLAAVRAAGRRDALRVTT..GREVIEL.RPDLDWDKGKTLRWVIDHLHRAG....SGSLTPVYLGDDI
41409576|M.avium              GEVAAAVRAAGRHDALRVTT..GREVIEL.RPDLDWDKGKTLHWVIEHLRRSG....SGALTPVYLGDDI
```

```
170575365|Brugia              η2  α12  β13  α13  β14
                                                 430       440       450       460       470       480
170575365|Brugia              SDI..............PMVRQAVKQNPDGVLAIFVGAKMSLREEVKQVIGDESRCCFVSCPDVIHAAMS
324509632|Ascaris             SDL..............PMVRQAVARNPEGVMALFVGVNENLRESVRQLVGDVSRCCFVSCPDVIHAAMA
402593175|Wuchereria          SDI..............PMVRQAVKQNPDGVLAIFVGAKMSLREEVKQVIGDVSRCCFVSCPDVIHAAMS
17568475|C.elegans            SDV..............PMLKKAADENPENVRALFVNINKQLQENITNIVGDAKRVCFISSPDVAHAAFA
308488095|C.remanei           SDV..............PMLQKAAEENPDEVRALFVNVSMELQKTIVNIVGDTSRTCFISCPDVAHAAFA
341884551|C.brenneri          SDV..............PMLQRAADENPNEVRALFVNVNEALQKTIGNIVGDPSRVCFISCPDVAHAAFA
268581923|C.briggsae          SDL..............PMLQKAAQENPTQVRALFVNVNKEIQSTINKIVGDSSRTCFISCPDVAHAAFA
339235773|Trichinella         SDL..............PMVQYAIQANRAGTIVTFVSPPAEVMQFLVET.ARPDGYCIVGCPEVIHVAMA
393906247|Loa                 SDI..............PMVRQAAKQNPDGVLTIFVGAKMSLREEVKQVVGDESRCCFVSCPDIIHAAML
15610508|M.tuberculosis       TDEDAFDVVG..P.HGVPIVVRHTDDGDRATAALFAL......................DSPARV.AEFT
340628353|M.canetti           TDEDAFDVVG..P.HGVPIVVRHTDDGDRATAALFAL......................DSPARV.AEFT
15827129|M.leprae             TDEDAFDAVRHTDVGGIPIVVRHTEDGNRATAALFTL......................DSPMHV.SEFT
183981178|M.marinum           TDEDAFDAVG..P.NGVAIMVRHNEDGDRATAALFAL......................ESPARV.AEFT
443489644|M.liflandii         TDEDAFDAVG..P.NGVAIMVRHNEDGDRATAALFAL......................ESPARV.AEFT
118616688|M.ulcerans          TDEDAFDAVG..P.NGVAIMVRHNEDGDRATAALFAL......................ESPARV.AEFT
495048542|M.colombiense       TDEDAFDAVRAGTVRGVPILVRHGDDGDRATAALFAL......................DSPTRA.AEFT
379756214|M.intracellulare    TDEDAFDAVH..D.DGVPILVRHSDDGDRATAALFAL......................DSPARA.AAFT
41409576|M.avium              TDEDAFDAVRGGPVQGVPILVRHNDDGDRATAALFAL......................DSPARA.AEFT
```

	α14
170575365|Brugia	
	490
**170575365|Brugia**	.QILNEH....CIGK
**324509632|Ascaris**	.ELLNEK....RATE
**402593175|Wuchereria**	.QILNEH....CIDR
**17568475|C.elegans**	.QIISE......FSG
**308488095|C.remanei**	.QIICE......LAA
**341884551|C.brenneri**	.QIICE.......HP
**268581923|C.briggsae**	.QIIIEL....TQNA
**339235773|Trichinella**	.RVLRKC...GALTT
**393906247|Loa**	.QILNEK.....CIG
**15610508|M.tuberculosis**	DRLARQLREAPLRAT
**340628353|M.canetti**	DRLARQLREAPLRAT
**15827129|M.leprae**	ERLARQLS...DTQR
**183981178|M.marinum**	GRLASQLS....TLG
**443489644|M.liflandii**	GRLASQLS....TLG
**118616688|M.ulcerans**	GRLASQLS....TLG
**495048542|M.colombiense**	GRLADQ.......LS
**379756214|M.intracellulare**	DRLADQL......EA
**41409576|M.avium**	ERLADQLE...RGEG
