# Supplementary material for: Structure of the Trehalose-6-phosphate Phosphatase from Brugia malayi Reveals Key Design Principles for Anthelmintic Drugs
Source: PLoS Pathog. 2014 Jul 3;10(7):e1004245. doi: 10.1371/journal.ppat.1004245 (PMC4081830; doi:10.1371/journal.ppat.1004245)
Supplement: Figure S6 — Sequence alignments of T6PP enzymes from a range of organisms. A sequence alignment of selected T6PP enzymes from a range of organisms reveals that the MIT-like domain is found only in nematodes and the Mycobacterium genus. (PDF) [file ppat.1004245.s006.pdf]

```
B.malayi
                1         10                      20          30          40          50
B.malayi        MTE..TVTDQGK.QRSSK.LQKN.....EAAKDEQVEGKGKETLESGTDKSAEQNSSLLV..GQPDVIDN
M.tuberculosis  MRKLGPVTIDPR.RHDAVLFDTTLDATQELVRQLQEVGVGTGVFGSGLDVPIVAAGRLAVRPGRCVVVSA
S.cerevisiae    MAL..KLDKEEKSNLESK.LWK.................EVPTI..........................
E.coli          MTE..PLTE.............................................................
T.acidophylum   M.....................................................................

                                                      α1                       α2
B.malayi
                60                                    70         80         90         100
B.malayi        DNV......................QTVDDFKNLMYKMQETRRAIVFALLNEKDLTKDDVEILKRAYEK
M.tuberculosis  HSAGVTAARESGFALIIGVDRTGCRDALRRDGADTVV..........TDLS.................
S.cerevisiae    ..........................QDWTNKFL...........SSLK.................
E.coli          ......................................................................
T.acidophylum   ......................................................................

                          β1      β2            α3                 TT     α4        α5
B.malayi
                 110         120         130         140         150         160         170
B.malayi        LTDNQTHSFQREMCTLTTKLSVNIGDETRGLEKDLKLYLDALMNIRREEPNLLWPIIMSRVDLFSILANYH
M.tuberculosis  ..................EVSVRTGDRRM...............SQLPDALQALG..............
S.cerevisiae    ..................EQASSDDDME...............RKMTPALNRPVL..............
E.coli          ......................................................................
T.acidophylum   ......................................................................

                            α6                 α7         β3            TT          α8
B.malayi
                 180         190         200         210         220         230         240
B.malayi        PKGKETFLKEYEDTVKFLKTFISSEAITGKKPIFITDWDGTMKDYCSQYATN.LQPVYSAVGMTRFAASF
M.tuberculosis  ......................LADGLVARQPAVFFDFDGTLSDIVEDPDAAWLAPG........ALEAL
S.cerevisiae    ......................LENYKQAKRRLFLFDYDGTLTPIVKDPAAAIPSAR........LYTIL
E.coli          .........................TPELSAKYAWFFDLDGTLAEIKPHPDQVVVPDN........ILQGL
T.acidophylum   ..............................IFLDYDGTLVPIIMNPEESYADAG........LLSLI

                    β4                  TTT         β5   η1   β6   TT   β7            α9
B.malayi
                  250         260         270         280         290         300         310
B.malayi        TRISAV..LTAGPLRGPGILDLTAMPI..DGPVMFSGSWGREWWLSGKRVVHQDGITDE.GFNALQRLDD
M.tuberculosis  QKLAAR..CPIAVLSGRDLADVTQRVG..LPGIWYAGSHGFELT.APDGTHHQNDAAAA.AIPVLKQAAA
S.cerevisiae    QKLCADPHNQIWIISGRDQKFLNKWLGGKLPQLGLSAEHGCFMK.DVSCQDWVN.LTEKVDMSWQVRVNE
E.coli          QLLATASDGALALISGRSMVELDALAK..PYRFPLAGVHGAERR.DINGKTHIVHLPDA.I...ARDISV
T.acidophylum   SDLKER..FDTYIVTGRSPEEISRFLP...LDINMICYHGACS..KINGQIVYNNGSDR.FLGVFDRIYE

                                             β8     β9              α10               β10
B.malayi
                        320         330         340         350         360         370         380
B.malayi        EMKDLLHTSDYAPFALVGSGVQRKVDRLTLGVQTVCHHVTSELSNRYQMAVKERMHRVDPNSQILVFDPS
M.tuberculosis  ELRQQLGP.....F..PGVVVEHKRFGVAVHY....RNAARD....RVGEVAAAVRTAEQRHALRVTTGR
S.cerevisiae    VMEEFTTR.....T..PGSFIERKKVALTWHY....RRTVPELGEFHAKELKEKLLSFTDDFDLEVMDGK
E.coli          QLHTVIAQ.....Y..PGAELEAKGMAFALHY....RQAPQH..EDALMTLAQRITQI..WPQMALQQGK
T.acidophylum   DTRSWVSD.....F..PGLRIYRKNLAVLYHL....GLMGAD....MKPKLRSRIEEIARIFGVETYYGK

                      β11                        α11                      β12      η2     α12
B.malayi
                        390         400                         410         420         430
B.malayi        TELEVEVVAHNSGIIWNKGNGVERL...............IKSLGDSLQSPGKILICGDTLSDIPMVRQA
M.tuberculosis  EVIELRPDV.....DWDKGKTLLWV...............LDHLPHSGSAPLVPIYLGDDITDEDAFDVV
S.cerevisiae    ANIEVRPRF......VNKGEIVKRLVWHQHGKPQDMLKGIREKLPK.DEMPDFVLCLGDDFTDEDMFRQL
E.coli          CVVEIKPRG......TSKGEAIAAF...............MQEAPFIGRT..PVFLGDDLTDESGFAVV
T.acidophylum   MIIELRVPG......VNKGSAIRSV...............RGE.....RP..AIIAGDDATDEAAFEAN

                                         β13                          α13
B.malayi
                  440                                     450                     460
B.malayi        VKQNP..................DGVLAIFV.....GAK.........MSLR...EEVKQVIGD.ESR
M.tuberculosis  ...GP..................HG.VPIVVRHTDDGDRATAALFALDSPARVA...EFTD.RLAR....
S.cerevisiae    ...NTIETCWKEKYPDQKNQWGNYGFYPVTVG...SASKKTVAKAHLTDPQQVL...ETLG.LLVGDVS.
E.coli          ...NR..................LGGMSVKI.....GTGATQASWRLAGVPDVW...SWLE.MITT....
T.acidophylum   ...D...................DA.LTIKV.....GEGETHAKFHVADYIEMRKILKFIE.MLG.....

                 β14                       α14
B.malayi
                470                    480                   490
B.malayi        CCFVSC..........PDVIHAAMSQIL..........NEHCIGK
M.tuberculosis  ......................QLR..........EAPLRAT
S.cerevisiae    .LFQSAGTVDLDSRGHVKNSESSLKSKLASKAYVMKRSASYTGAKV
E.coli          ......................AL.QQKRENN..RSDDYESFSRSI
T.acidophylum   .....................................VQKKQ
```
