# Supplementary material for: Structure of the Trehalose-6-phosphate Phosphatase from Brugia malayi Reveals Key Design Principles for Anthelmintic Drugs
Source: PLoS Pathog. 2014 Jul 3;10(7):e1004245. doi: 10.1371/journal.ppat.1004245 (PMC4081830; doi:10.1371/journal.ppat.1004245)
Supplement: Table S1 — Elimination of tps-1 function suppresses the larval arrest caused by gob-1 RNAi. (DOCX) [file ppat.1004245.s010.docx]

**Table S1. Elimination of *tps-1* function suppresses the larval arrest caused by *gob-1* RNAi**

| Genotype | *gob-1* RNAi phenotype (n=45) | |
| --- | --- | --- |
| *eri-1(mg366); lin-15B(n744)* | | Larval arrest |
| *eri-1(mg366); lin-15B(n744); tps-1(ok373)* | | Wild-type |
