# Supplementary material for: Structure of the Trehalose-6-phosphate Phosphatase from Brugia malayi Reveals Key Design Principles for Anthelmintic Drugs
Source: PLoS Pathog. 2014 Jul 3;10(7):e1004245. doi: 10.1371/journal.ppat.1004245 (PMC4081830; doi:10.1371/journal.ppat.1004245)
Supplement: Table S2 — List of cloning and mutagenesis primers used in this study. (DOCX) [file ppat.1004245.s011.docx]

**Table S2. List of cloning and mutagenesis primers used in this study.**

| Construct or mutation |  | Sequence (mutated nucleotides in lowercase) |
| --- | --- | --- |
| Bm T6PP | F  R | 5’-GATCGGGAATTCCATATGACGGAAACGGTTACAGA  5’-GATCGGATCCTCATTTGCCGATGCAATGTTC |
| Bm T6PP Δ59 | F  R | 5’-GATAATGTTCAAACCGTTGATGATTTTAAGAATCTCATG  5’-ATGGCCCTGGAAATACAGGTTTTCGCC |
| Bm T6PP Δ179 | F  R | 5’-GGCAAAGAACATTTTTGAAGGAATATGAAGATACTG  5’-ATGGCCCTGGAAATACAGGTTTTCGCC |
| D213A | F  R | 5’-gcgTGGGATGGTACAATGAAAGACTATTGTTCGC  5’-CGTGATAAATATTGGTTTCTTACCGGTAATCG |
| D215A | F  R | 5’-gctGGTACAATGAAAGACTATTGTTCGCAATATGC  5’-CCAATCCGTGATAAATATTGGTTTCTTACCGG |
| Y221A | F  R | 5’-gcgTGTTCGCAATATGCAACAAATCTTCAGCC  5’-GTCTTTCATTGTACCATCCCAATCCGTGATAA |
| Y225A | F  R | 5’-gcgGCAACAAATCTTCAGCCTGTGTATAGTGC  5’-TTGCGAACAATAGTCTTTCATTGTACCATCCC |
| N228A | F  R | 5’-TATGCAACAgctCTTCAGCCTGTGTATAGTGCAG  5’-TTGCGAACAATAGTCTTTCATTGTACCATCCC |
| W280A | F  R | 5’-gcgGGTCGTGAATGGTGGCTATCTGGAAAAC  5’-AGAACCGCTGAACATTACAGGACCATCA |
| S329A | F  R | 5’-gctGGTGTGCAACGAAAAGTGGATAGATTAACG  5’-TCCAACAAGTGCAAATGGAGCGTAATC |
| Q332A | F  R | 5’-gcaCGAAAAGTGGATAGATTAACGTTAGGTGTCC  5’-CACACCACTTCCAACAAGTGCAAATGGA |
| K334A | F  R | 5’-gcaGTGGATAGATTAACGTTAGGTGTCCAAACTGT  5’-TCGTTGCACACCACTTCCAACAAGTG |
| D336A | F  R | 5’-cgAGATTAACGTTAGGTGTCCAAACTGTTTGCC  5’-CCACTTTTCGTTGCACACCACTTCCAAC |
| R337A | F  R | 5’-gcaTTAACGTTAGGTGTCCAAACTGTTTGCCAT  5’-ATCCACTTTTCGTTGCACACCACTTCC |
| T339A | F  R | 5’-gcgTTAGGTGTCCAAACTGTTTGCCATCACG  5’-TAATCTATCCACTTTTCGTTGCACACCACT |
| D378A | F  R | 5’-gctCCATCCACAGAATTAGAAGTTGAAGTAGTTGC  5’-AAATACCAAAATCTGACTGTTAGGATCTACGC |
| E384A | F  R | 5’-gcgGTTGAAGTAGTTGCACACAATTCGGGAATAAT  5’-TAATTCTGTGGATGGATCAAATACCAAAATCTGAC |
| E386A | F  R | 5’-gcgGTAGTTGCACACAATTCGGGAATAATATGGAAT  5’-AACTTCTAATTCTGTGGATGGATCAAATACCAAAATC |
| L229E | F  R | 5’-gaaCAGCCTGTGTATAGTGCAGTTGGAATGAC  5’-ATTTGTTGCATATTGCGAACAATAGTCTTTCAT |
| L229Y  V236S | F  R | 5’-CACAGGCTGAtaATTTGTTGCATATTGCGAACAATAG  5’-TATAGTGCAtcTGGAATGACACGATTCGCAGCTTC |
| V232S  A242K | F  R | 5’-TCCAGATGCACTATAgctAGGCTGATAATTTGTTGCATATTGC  5’-ATGACACGATTCGCAaaaTCTTTTACTCGTATTAGCGCAGTGT |
