# Supplementary material for: Structure of the Trehalose-6-phosphate Phosphatase from Brugia malayi Reveals Key Design Principles for Anthelmintic Drugs
Source: PLoS Pathog. 2014 Jul 3;10(7):e1004245. doi: 10.1371/journal.ppat.1004245 (PMC4081830; doi:10.1371/journal.ppat.1004245)
Supplement: Table S3 — Crystallographic and refinement statistics for B. malayi T6PP data sets. (DOCX) [file ppat.1004245.s012.docx]

**Table S3. Crystallographic and refinement statistics for *B. malayi* T6PP data sets**

|  | Data Set | | |  |  |
| --- | --- | --- | --- | --- | --- |
| Parameters | **Native** | **SelenoMet Derivative** | | |  |
| Space group | P6_4_22 | | P6_4_22 |  |  |
| Unit cell dimensions (Å) | 166.94 x 166.94 x 99.56 | | 164.88 x 164.88 x 92.62 |  |  |
| Cell angles (°) | 90 x 90 x 120 | | 90 x 90 x 120 |  |  |
| Solvent content (%) | 67.3 | | 62.7 |  |  |
| Vm (Å^3^/Dalton) | 3.8 | | 3.3 |  |  |
| Molecules per ASU | 1 | | 1 |  |  |
| Wavelength (Å) | 0.9795 | | 0.9795 |  |  |
| I/σ (total/highest) | 24.34 (3.48) | | 21.33 (1.67) |  |  |
| Completeness (total/highest) | 99.9 (99.6) | | 99.9 (99.70) |  |  |
| Multiplicity (total/highest) | 10.6 (9.9) | | 37.8 (35.4) |  |  |
| R_merge_ (total/highest) | 0.092 (0.94) | | 0.163 (0.75) |  | |
| Anomalous signal | n/a | | 0.111 |  |  |
| Practical resolution limit of anomalous signal (Å) | n/a | | 4.87 |  |  |
| Resolution range (Å) | 20.00 – 2.90 (2.95 – 2.90) | | 25.00 – 3.10 (3.15 – 3.10) |  |  |
|  | **Refinement statistics for the native dataset** | | |  |  |
| Resolution range (Å) | 19.91 – 3.00 (3.08 – 3.00) | | |  |  |
| Unique reflections | 16,791 | | |  |  |
| Test set | 1,679 (10%) | | |  |  |
| R_work_ | 0.215 | | |  |  |
| R_free_ | 0.259 | | |  |  |
| Mean B value (Å^2^)  macromolecule  ligands  solvent | 81.5  81.6  55.6  63.4 | | |  |  |
| Total waters | 85 | | |  |  |
| RMSD  Bond length (Å)  Bond angle (°) | -  1.16  0.005 | | |  |  |

**Supplemental Table 3. Crystallographic and refinement statistics for *B. malayi* T6PP data sets (continued)**

| Model building  Residues modeled  Residues omitted | 63-110, 114-319, 323-366, 371-491  1-62, 111-113, 320-322, 367-370, 492 |
| --- | --- |
| Ramachandran plot  Core region  Additional allowed region  Outliers | 382 (93.2)  27 (6.6%)  1 (0.2%) |
